# Morphological Changes within the Rat Lateral Ventricle after the Administration of Proteasome Inhibitors

**DOI:** 10.1371/journal.pone.0140536

**Published:** 2015-10-19

**Authors:** Sławomir Wójcik, Jan Henryk Spodnik, Jerzy Dziewiątkowski, Edyta Spodnik, Janusz Moryś

**Affiliations:** Department of Anatomy and Neurobiology, Medical University of Gdańsk, Gdańsk, Poland; University of Palermo, ITALY

## Abstract

The broad variety of substances that inhibit the action of the ubiquitin-proteasome system (UPS)—known as proteasome inhibitors—have been used extensively in previous studies, and they are currently frequently proposed as a novel form of cancer treatment and as a protective factor in intracerebral hemorrhage treatment. The experimental data on the safest route of proteasome inhibitor administration, their associated side effects, and the possible ways of minimizing these effects have recently become a very important topic. The aim of our present study was to determine the effects of administering of MG-132, lactacystin and epoxomicin, compounds belonging to three different classes of proteasome inhibitors, on the ependymal walls of the lateral ventricle. Observations were made 2 and 8 weeks after the intraventricular administration of the studied substances dissolved in dimethyl sulfoxide (DMSO) into the lateral ventricle of adult Wistar rats. Qualitative and quantitative analysis of brain sections stained with histochemical and inmmunofluorescence techniques showed that the administration of proteasome inhibitors caused a partial occlusion of the injected ventricle in all of the studied animals. The occlusion was due to ependymal cells damage and subsequent ependymal discontinuity, which caused direct contact between the striatum and the lateral nuclei of the septum, mononuclear cell infiltration and the formation of a glial scar between these structures (with the activation of astroglia, microglia and oligodendroglia). Morphologically, the ubiquitin-positive aggregates corresponded to aggresomes, indicating impaired activity of the UPS and the accumulation and aggregation of ubiquitinated proteins that coincided with the occurrence of glial scars. The most significant changes were observed in the wall covering the striatum in animals that were administered epoxomicin, and milder changes were observed in animals administered lactacystin and MG-132. Interestingly, DMSO administration also caused damage to some of the ependymal cells, but the aggresome-like structures were not formed. Our results indicate that all of the studied classes of proteasome inhibitors are detrimental to ependymal cells to some extent, and may cause severe changes in the ventricular system. The safety implications of their usage in therapeutic strategies to attenuate intracerebral hemorrhagic injury and in brain cancer treatment will require further studies.

## Introduction

The ubiquitin-proteasome system (UPS), the main system responsible for protein degradation in eukaryotic cells, has currently become the topic of very intensive research [1]. The results of these studies helped to establish the relationships between UPS activity and neurodegenerative pathogenesis during aging, the inflammatory response, and the dynamics of tumour development [2, 3]. A broad variety of substances that inhibiting UPS activity—proteasome inhibitors—were used in previous studies (e.g., as an experimental model of neurodegenerative diseases) and are currently frequently proposed as a form of cancer treatment for solid tumours and haematological malignancies, and may be novel therapeutic strategies for ischemia-reperfusion injury in the brain [1, 3–7]. Treatments for CNS disorders are still a very complex therapeutic problem, due to organ specificity. The restrictions on the use of surgical therapy force us to use drug therapy, often polytherapy with proteasome inhibitors.

Proteasome inhibitors are a very diverse group in terms of their chemical structures and their biological actions (for review see [1]). Therefore, the research on the safe route of administration, their side effects on healthy nervous tissue, and the possible ways of minimizing these effects has recently become a very important topic. There is data from in vivo studies about the influence of proteasome inhibitors administered intraperitoneally [8, 9], subcutaneously [10], into the ventricular system [11] or after direct injection into selected brain structures [12–14] on cellular subpopulations in the nervous tissue. However, there is currently no data available about the influence of proteasome inhibitors on ependymal cells. The aim of this study is to determine the changes that occur in the walls of the rat lateral ventricle after intraventricular administration of selected covalent proteasome inhibitors—MG-132 (an example of peptide aldehydes), lactacystin (an example of β-lactones and derivatives) and epoxomicin (an example of epoxyketones).

## Materials and Methods

### Ethical statement

In this study, the rats’ care and treatment was performed in strict accordance with the recommendations given in the “Guide for the care and use of laboratory animals” of the Polish National Committee on the Ethics of Animal Experiments. The experimental procedures were reviewed and approved by the 3^rd^ Local Committee on the Ethics of Animal Experiments in Gdansk (Local Committee of the Medical University of Gdansk—Permit Number: 08–2007). All efforts were made to reduce the number of animals and to minimize their suffering.

### Animals

Forty adult (postnatal day 90) male Wistar rats (initial weight between 230 and 270 g) were used in this study. All rats were bred until they reached the appropriate age in the Tri-City Academic Laboratory Animal Centre—Research and Services Centre. All of the animals were bred behind a sanitary-hygienic barrier, which is supported by HEPA filters. The rats were kept in air-conditioned spaces under a constant temperature (22±2°C) and humidity (55±10%), with a defined lighting regimen (lights on from 7:00 a.m. to 7:00 p.m.). All rats were housed socially in polysulfone cages (size T. IV) with an area of 1875 square centimetres and a height of 195 mm (3–4 animals per cage) strewn with powderless sawdust from deciduous trees. The rats were fed a standard food mixture for rodents, which is manufactured by SSNIFF (Spezialdiäten GmbH, Germany). Both food and water were autoclaved before being served to the animals and water was available ad libitum. All of the rats were supplied to the Department Animal Centre two weeks before our experiments began and were housed in similar conditions.

### Proteasome inhibitor administration and postoperative procedures

The animals were randomly divided into four groups. All rats were anesthetized with an intraperitoneal administration of fentanyl (1 mg/kg) and dehydrobenzperidol (0.2 mg/kg). The animals were fixed in a stereotaxic frame (Trend Wells, USA) under sterile conditions, and after an incision in the skin, a small craniectomy was made above the selected cortical area. The cerebral cortex was exposed after an incision of the dura mater. A glass micropipette coated with silicone gel was inserted through the cerebral cortex into the right lateral ventricle, based on the stereotaxic coordinates from the bregma point: 0.6 mm posteriorly, 1.5 mm laterally, and 3.8 mm ventrally [15]. Pressure injections were made using a 5 μl Hamilton syringe at a rate of 0.8 μl/min. The first group, which is referred to as the controls (n = 10) in the subsequent text, consisted of animals that received a 4 μl injection of 100% dimethyl sulfoxide (DMSO; Sigma-Aldrich, St Louis, MO). The second group is referred to as MG-132 (n = 10), the third group as lactacystin (n = 10) and the fourth group as epoxomicin (n = 10) consisted of animals that received injections of 32 μg MG-132 (Boston Biochem Inc.; cat # I-130.5, lot # 3570428), 20 μg lactacystin (Boston Biochem Inc.; cat # I-115) or 4 μg epoxomicin (Boston Biochem Inc.; cat # I-110), respectively; all proteasome inhibitors were dissolved in 4 μl of DMSO. The doses administered to the animals were based on those previously reported in the literature [11, 16]. After each injection, the micropipette was held in place for at least 5 min to prevent leakage of the administered substance and then gently retracted, and the wound was surgically closed. The experimental endpoints have been set at two and eight weeks after the surgery. None of the animals died prior to the experimental endpoint. During the post-operative period (2 days), all animals were monitored for their physical conditions twice a day by technician from the animal lab (7:10 a.m. and 6:50 p.m.), and also twice a day at random times by the chief researcher. Later, all animals were monitored for their physical conditions twice a day by technician from the animal lab (7:10 a.m. and 6:50 p.m.), and also once a day at a random time by the chief researcher. The food/water intake were noted, as well as clinical signs of pain or discomfort, such as weight loss, body condition score, inappetence, weakness/inability to obtain water or food, moribund state, signs of infection and signs of severe organ dysfunction (non-responsive to treatment), such as dyspnoea, blood loss, diarrhoea or obstruction, CNS depression, seizures, paralysis of one or more extremities, muscle damage, bone injury, and non-healing wounds. The evaluations of the body weight and basic body condition scores were performed according to the recommendations by Ullman-Cullere and Foltz [17]. According to the protocol based on the Guidelines for Endpoints in Animal Study Proposals by the Office of Animal Care and Use (revised– 11/14/07) [18] and the OECD Guidance Document on the Recognition, Assessment, and Use of Clinical Signs as Humane Endpoints for Experimental Animals Used in Safety Evaluation [19], the animals underwent veterinary care in the event that a problem was detected. If the animal required euthanasia, decapitation after anaesthesia was planned. None of the animals required this procedure prior to the experimental endpoint.

### Tissue collection and preparation

Two weeks (half of the animals from each studied group) or eight weeks (the second half of the animals from each group) after intraventricular administration of the studied substances, the rats were deeply anaesthetized with lethal doses of intraperitoneal injections of Nembutal (80 mg/kg of body weight). The rats were transcardially perfused with 0.9% saline containing 10,000 units of Heparin, followed by a 4% solution of paraformaldehyde in 0.1 M phosphate buffer (pH 7.4; 4°C). Directly after perfusion, the brains were removed from the skulls, postfixed in 4% paraformaldehyde for 3–4 hours, and then cryoprotected initially in 10% (overnight at 4°C) and subsequently in a 30% phosphate-buffered sucrose solution (pH 7.4; 4°C), until the tissues sank. Before sectioning, each left hemisphere was marked by slight damage of the neocortex, and then the brains were cut on a cryostat (Microm HM 525, Thermo Scientific) into 40 μm thick coronal sections. Every sixth pair of sections was saved, mounted and stained using the cresyl violet method (Nissl staining). The sections were coverslipped with DPX (Fluka, Germany) and studied under an optical Labophot-2 microscope (Nikon, Japan). For the subsequent studies, the remaining sections were collected and preserved in a cryoprotective solution containing glycerol.

### Immunofluorescence and histofluorescence

To evaluate the possible changes in the structures neighboring the lateral ventricle following proteasome inhibitor administration, immunofluorescence staining was performed with the following primary antibodies: anti-ubiquitin (Abcam; Cat. No. ab7780), anti-GFAP (Novocastra™ Liquid Mouse Monoclonal Antibody Glial Fibrillary Acidic Protein, Product Code: NCL-L-GFAP-GA5, lot 6006116), anti-NogoA (Santa Cruz Biotechnology; Cat. No. sc-25660), and OX-42 (Serotec; Cat. No. MCA275G). GFAP (glial fibrillary acidic protein) is a well-established marker of astroglia [20], while NogoA (Neurite outgrowth inhibitor reticulon-4) is proposed to be a reliable marker of oligodendrocytes [21]. The OX-42 monoclonal antibody, which recognizes the rat equivalent of human CD11b, the iC3b complement receptor, is a marker of microglia [22]. The staining protocol was consistent with a previously described protocol [9]. Briefly, free-floating sections were blocked in 3% normal goat serum (NGS) and 0.3% Triton X-100 in 0.01 M PBS (pH 7.2) for 1 hour at room temperature. Next, they were incubated with the indicated primary antibodies in 0.01 M PBS (pH 7.2) containing NGS and 0.1% Triton X-100 at 4°C. After 48 hours, the sections were washed with PBS and incubated (2 hours at room temperature) with a secondary goat anti-mouse antibody coupled with Alexa Fluor^®^ 488 (Life Technologies, Cat. No. A-11001, lot 481679; 1:500) and a goat anti-rabbit antibody coupled with Alexa Fluor^®^ 647 (Life Technologies, Cat. No. A-21109; 1:500). Then, the sections were stained with NeuroTrace^®^ 530⁄615 Red Fluorescent Nissl Stain (Life Technologies, Cat. No. N-21482; lot 461322) according to the manufacturer's protocol. Finally, the sections were washed with PBS, mounted onto gelatin-coated slides, air-dried and coverslipped with Keiser Gelatin (Merck, Germany). The omission of the primary antibodies during the control experiments resulted in a lack of signal.

### Qualitative and quantitative analyses

The initial analysis of stained sections (the images up to 200X magnification) was performed on an MVX10 MacroView Fluorescence Microscope (Olympus, Japan) equipped with an XC50 digital camera (Olympus, Japan). The high-magnification images and co-localization study were performed using a confocal laser scanning microscopy (CLSM) system (Radiance 2100, Bio-Rad UK) mounted on a microscope (Eclipse 600, Nikon, Japan). The CLSM images were obtained with 40X and 60X oil immersion objective lenses of N.A. = 1.3 and 1.4, respectively. The optimal iris diameter was used for each magnification. The CLSM images were analyzed with the LaserSharp 2000 and LaserPix v. 2.0 software (both Bio-Rad, UK).

To estimate the extent of adhesion between the striatum and septum (glial scar) in each rat, all Nissl-stained serial coronal sections were compared to the atlas images [15], and the distance (in mm) from bregma point in the sagittal axis was calculated. After determining the length of the adhesion and establishing that the adhesion can reach a maximum in a section located 1.6 mm anteriorly and 0.8 mm posteriorly from the bregma point, the major morphological alterations in the tissue were quantitatively analyzed by light microscopy at 10x and 40x magnifications. In the first step, five systematically and randomly chosen coronal sections of the brain were taken from 1.7 mm anterior to 1.0 mm posterior from the bregma point from each animal [15]. On those sections, the following parameters were estimated within the wall of the lateral ventricle and subventricular area: the presence and localisation of ependymal discontinuity, the presence and number of apoptotic bodies per section, the presence of ependymal atrophy, the presence of a glial scar, the presence of ependymal rosettes, the presence of glial nodules, and the presence of mononuclear inflammatory cell infiltrations. Additional scoring of the ependymal discontinuity and mononuclear inflammatory cell infiltrations observed in the rat brain coronal sections was performed. They were scored as follows: none, mild, moderate, and high degree.

### Statistical analysis

The statistical analysis was performed using the data analysis software system STATISTICA version 12, StatSoft Inc. (2014).

The frequency of ependymal atrophy, apoptosis, ependymal discontinuity and rosettes, glial nodules and inflammatory cell infiltration in the animals from the various experimental groups sacrificed 2 weeks and 8 weeks after studied substances application was calculated. A 2x2 Fisher test was utilized to compare these frequencies for each substance. The level of significance was set to 0.05. The differences between the control (DMSO) group and each of the other experimental groups (MG-132 or lactacystin or epoxomicin) were also compared at 2 weeks and 8 weeks using the 2x2 Fisher test by taking into account the experiment-wise error rate and applying Šidak’s correction [23] for multiple comparisons. The level of significance was set to 0.017. The frequency data in Table 1 are expressed as percentages.

**Table 1:** Summary of the quantification of the morphological alterations observed after intraventricular administration of DMSO and the proteasome inhibitors.

Morphological alteration		2 weeks				8 weeks			
		DMSO	MG-132	Lactacystin	Epoxomicin	DMSO	MG-132	Lactacystin	Epoxomicin
**Degree of ependymal discontinuity** [frequency in %]	none	64	8	20	8	32	32	20	20
	mild	24	28	44	4	24	40	8	16
	moderate	12	44	24	24	32	16	56	28
	high	0	20	12	64	12	12	16	36
**Localization of ependymal discontinuity** [frequency in %]	septum	32	92	72	92	64	60	80	80
	striatum	8	48	40	32	24	20	20	28
	corpus callosum	12	20	7	8	44	32	36	40
**Apoptotic bodies** [frequency in %]		56	100	84	80	76	96	92	96
**Apoptotic bodies** [n/section]		2.9 ± 0.3	3.6 ± 0.3	3.9 ± 0.2	5.7 ± 0.3	4.5 ± 0.9	4.6 ± 0.9	3.6 ± 0.2	7.3 ± 0.9
**Ependymal atrophy** [frequency in %]		28	52	48	84	52	60	96	94
**Glial scar** [length in sagittal axis in mm]		0.5 ± 0.2	1.0 ± 0.2	1.3 ± 0.3	1.3 ± 0.4	0.4 ± 0.4	1.2 ± 0.3	1.5 ± 0.8	1.8 ± 0.5
**Ependymal rosettes** [frequency in %]		0	16	0	0	16	40	24	32
**Glial nodules** [frequency in %]		16	24	20	40	14	16	12	20
**Degree of inflammatory cell infiltration** [frequency in %]	none	56	16	24	8	36	28	4	12
	mild	12	8	16	0	32	24	24	8
	moderate	20	48	28	36	28	44	72	40
	high	12	28	32	56	4	4	0	40

See the Materials and Methods for the details of the quantifications.

The distributions of the ependymal discontinuity and mononuclear inflammatory cell infiltration scores were compared. The differences between the control (DMSO) group and each of the other experimental groups (MG-132 or lactacystin or epoxomicin) were compared at 2 weeks and 8 weeks using the Chi-square test, also taking into account the experiment-wise error rate and applying Šidak’s correction for multiple comparisons. The level of significance was set to 0.017. The scoring distribution data in Table 1 are expressed as percentages.

The differences in (1) the number of apoptotic bodies per section and (2) the length of the adhesions connecting the ventricular walls were compared between the experimental groups using two-way ANOVA, followed by Fisher’s post-hoc LSD test (upon normality checking using the Kolmogorov-Smirnov test). The differences were considered significant when p<0.05. The data in Table 1 are expressed as the means ± standard deviation (SD).

## Results

**Fig 1:**
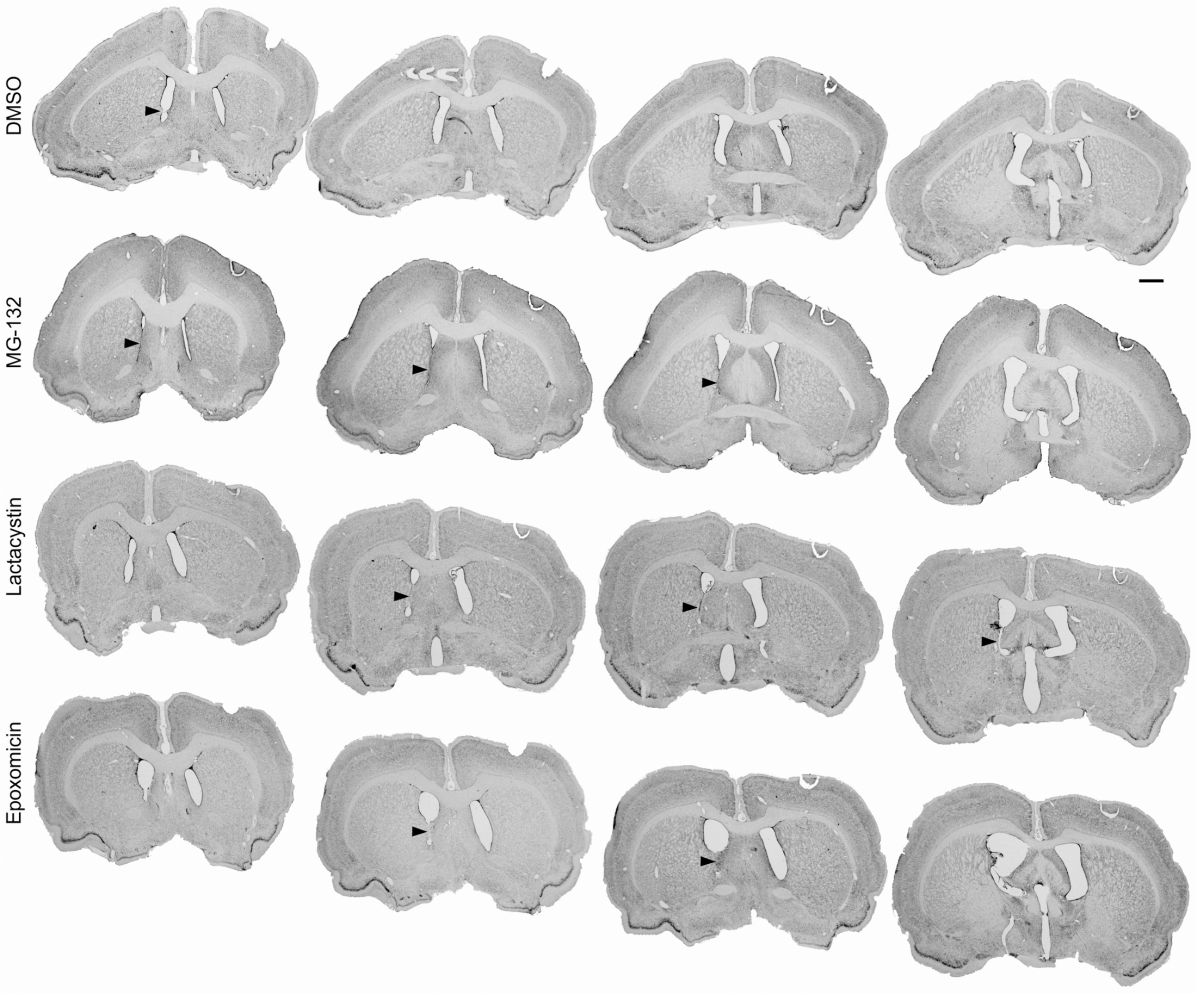
Partial occlusion of the rat lateral ventricle 2 weeks after administering the studied substances. Macroscopic view of the morphological changes within the right lateral ventricle of the rat using serial coronal sections of the rats’ brains at 2 weeks after the administration of the studied substances—DMSO, MG-132, lactacystin and epoxomicin. The arrows indicate the adhesion between the ventricular walls. Staining—Nissl method; scale bar = 2 mm.

**Fig 2:**
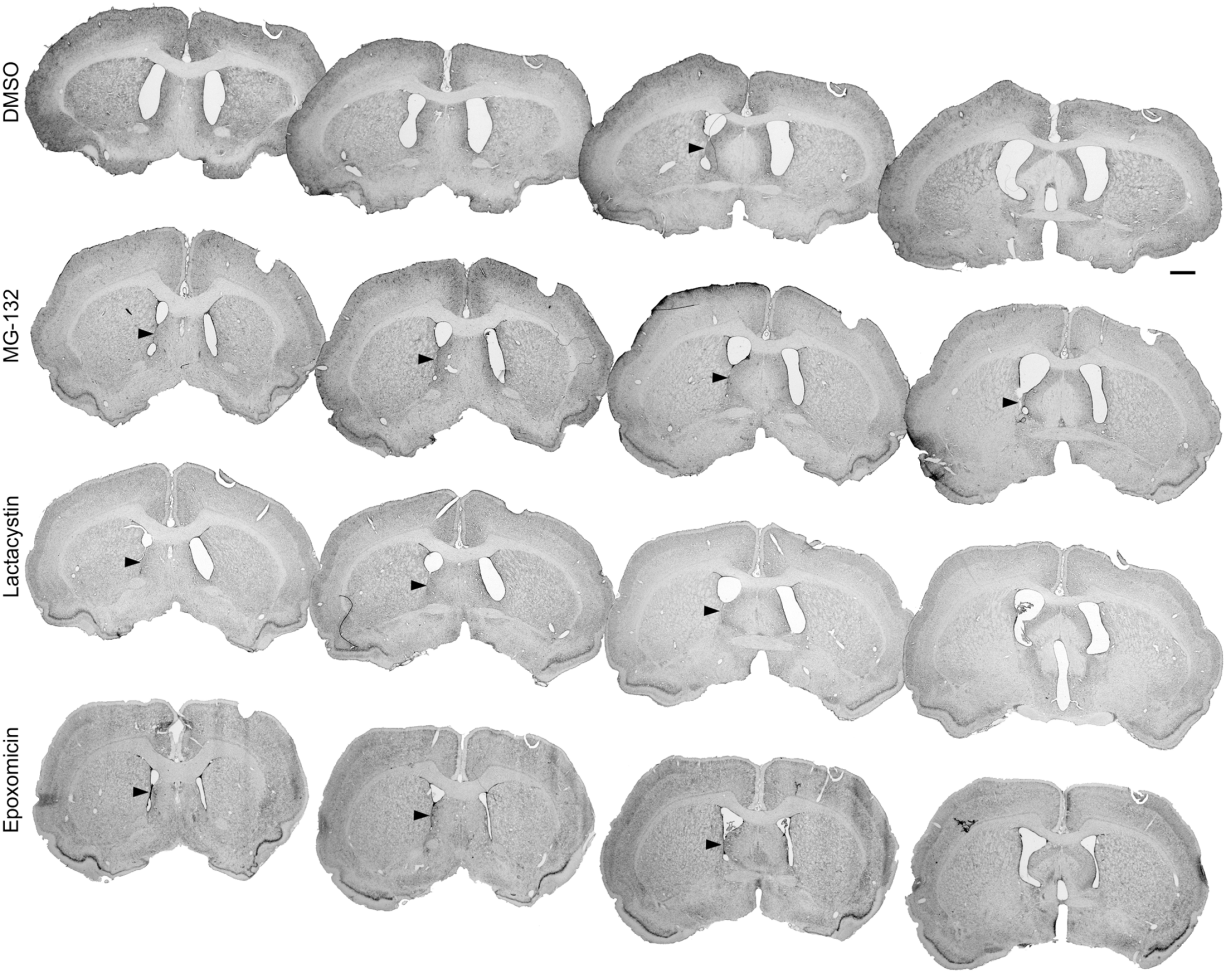
Partial occlusion of the rat lateral ventricle 8 weeks after administering the studied substances. Macroscopic view of the morphological changes within the right lateral ventricle of the rat using serial coronal sections of the rats’ brains at 8 weeks after the administration of the studied substances—DMSO, MG-132, lactacystin and epoxomicin. The arrows indicate the adhesion between the ventricular walls. Staining—Nissl method; scale bar = 2 mm.

In adult rats, experimental application of the studied proteasome inhibitors, MG-132, lactacystin and epoxomicin, as well as the DMSO solvent alone, caused injury with a subsequent ependymal reaction within the right lateral ventricle. The evaluation of the Nissl-stained sections revealed several morphological alterations: ependymal discontinuity, ependymal atrophy, subependymal gliosis, ependymal rosette formation and mononuclear inflammatory cell infiltrations present within ventricular walls and subventricular area on the injection side. In all animals treated with proteasome inhibitors, a combination of these morphological changes led to glial scar formation, resulting in partial occlusion of the injected ventricle (Figs 1 and 2). In the control group, which was given 4 μl of DMSO, partial occlusion of the lateral ventricle was present in 60% of the studied cases as soon as two weeks after application. Eight weeks after the DMSO application, occlusion was observed in 80% of cases. Evidence of intraventricular hemorrhage was not present in any of the animals from the groups sacrificed at 2 or 8 weeks after the surgery.

### Ependymal discontinuity

**Fig 3:**
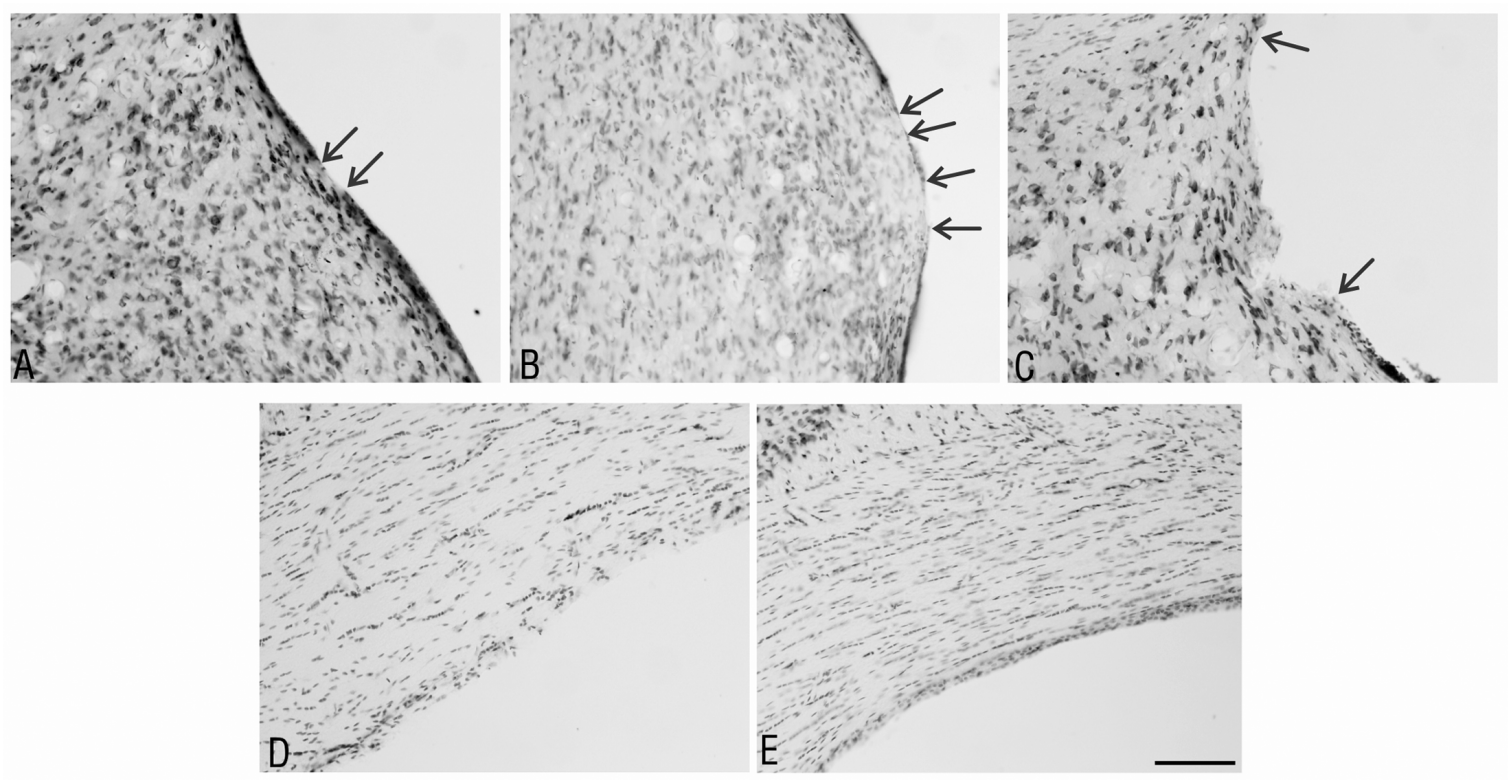
The characteristics of the ependymal discontinuity after intraventricular proteasome inhibitor administration. Microscopic view of the different degrees of ependymal discontinuity: (A) mild, (B) moderate, (C) high on the surface of the septum, and (D) high on the surface of the corpus callosum. (E) Normal control with an unchanged ventricular ependymal lining from the same section as (D), but from the opposite side. The arrows indicate the borders between the area of the ependymal discontinuity and the existing ependymal lining. The sections were from the the rat brains at 2 weeks after administration of: A—DMSO; B, C—MG-132; and D, E—epoxomicin. Staining—Nissl method; scale bar = 100 μm.

In all studied cases, the ventricular lining, which is comprised of ciliated, cuboidal ependymal cells, was damaged to different extents. The mildest degree of ependymal discontinuity was characterized by single, small areas lacking the ependymal lining. In cases with moderate damage, the ependyma-deprived areas were larger, more numerous and divided by the existing ependymal lining. In cases with the highest degree of damage, particularly in the animals treated with the proteasome inhibitors, many ependymal cells were detached from the ventricular walls, leaving its entire surface without protection (Fig 3).

Two weeks after the injection of the studied substances, the ependyma-deprived areas in the ventricular border were primarily observed in the proximity of the ventral half of the injected lateral ventricle. Those areas were mainly present on the surface of lateral septal nuclei and, to a lesser extent, on the surface of the striatum (Table 1).

Two weeks after the injection of DMSO, ependymal discontinuity was observed in 36% of the studied sections. At the same time point after the application of the proteasome inhibitors, the frequency of ependymal discontinuity was 2.6-fold higher in the MG-132-treated rats, 2.2-fold higher in the lactacystin-treated rats, and 2.6-fold higher in the epoxomicin-treated rats. Two weeks after the injection, the degree of ependymal discontinuity present in the sections (Table 1) was significantly more severe in the MG-132 (p<0.001), lactacystin (p = 0.011) or epoxomicin (p<0.001) groups than that after the DMSO application. Eight weeks after the injection of the studied substances, ependyma-deprived areas in the ventricular border were also observed at the ventral half of the injected lateral ventricle in the proximity of the formed glial scar but also at the top of the lateral ventricle. In contrast to the animals sacrificed 2 weeks after the injection of the studied substances, those areas comprised the surface of the lateral septal nuclei and the surface of the corpus callosum in the animals sacrificed 8 weeks after the injection (Table 1). Eight weeks after the injection of DMSO, ependymal discontinuity was observed in 68% of the studied sections, which was significantly more often (p<0.05) than that after two weeks. At eight weeks, the frequency of the ependymal discontinuity observed in the MG-132-treated rats was the same, but was similar in the lactacystin- and epoxomicin–treated rats (Table 1). Statistical analysis showed that at eight weeks after the injection, there were no significant differences between the degree of ependymal discontinuity in the sections from the animals injected with DMSO or any of the studied proteasome inhibitors.

**Fig 4:**
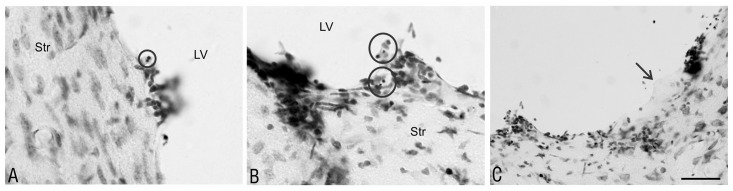
Morphological hallmarks of apoptosis and subependymal gliosis after intraventricular proteasome inhibitor administration. Apoptotic bodies (encircled) and glial tubercles (arrow) were present in the ependymal lining of the striatum (Str) 2 weeks after MG-132 (A) and epoxomicin (B, C) administration into the right lateral ventricle (LV). Staining—Nissl method; scale bars: A, B—25 μm; C—50 μm.

Ependymal discontinuity was related to another of the observed morphological alterations of the ependymal cells. The nuclei were compacted in some cells and morphological markers of apoptotic cell death, apoptotic bodies, were visible (Fig 4).

Two weeks after the application of the studied substances, the frequency of the sections with apoptotic bodies was slightly higher after the proteasome inhibitor application: 1.8-fold higher in the MG-132-treated rats, 1.5-fold higher in the lactacystin-treated rats, and 1.4-fold higher in the epoxomicin-treated rats compared to the DMSO-treated rats (Table 1). At this time, the density of apoptotic bodies in the sections from animals treated with epoxomicin was significantly higher than those treated with DMSO (p = 0.003) or MG-132 (p = 0.02) (Table 1). Eight weeks after the application of the studied substances, the frequency of sections with apoptotic bodies was very similar in all of the studied groups of animals (Table 1). However, at this time point, the density of apoptotic bodies in the sections from animals treated with epoxomicin was significantly higher than those treated with DMSO (p = 0.003), MG-132 (p = 0.004) or lactacystin (p<0.001) (Table 1).

### Ependymal atrophy

Based on the histopathology, ependymal atrophy is recognized when the ependymal cells become flattened and exhibit a loss of cytoplasm. Another characteristic feature of ependymal atrophy is the localization of the nucleus, which is most often adjacent to the main volume of the cytoplasm rather than basal to it [24]. The microvilli and cilia are usually reduced in number, but do not necessarily completely disappear. Two weeks after the injection of the studied substances, ependymal atrophy (ventricular walls lined by flat epithelioid cells devoid of cilia) was observed in the areas located in the proximity of the ventral half of the injected lateral ventricle on the surface of the septum and striatum.

**Fig 5:**
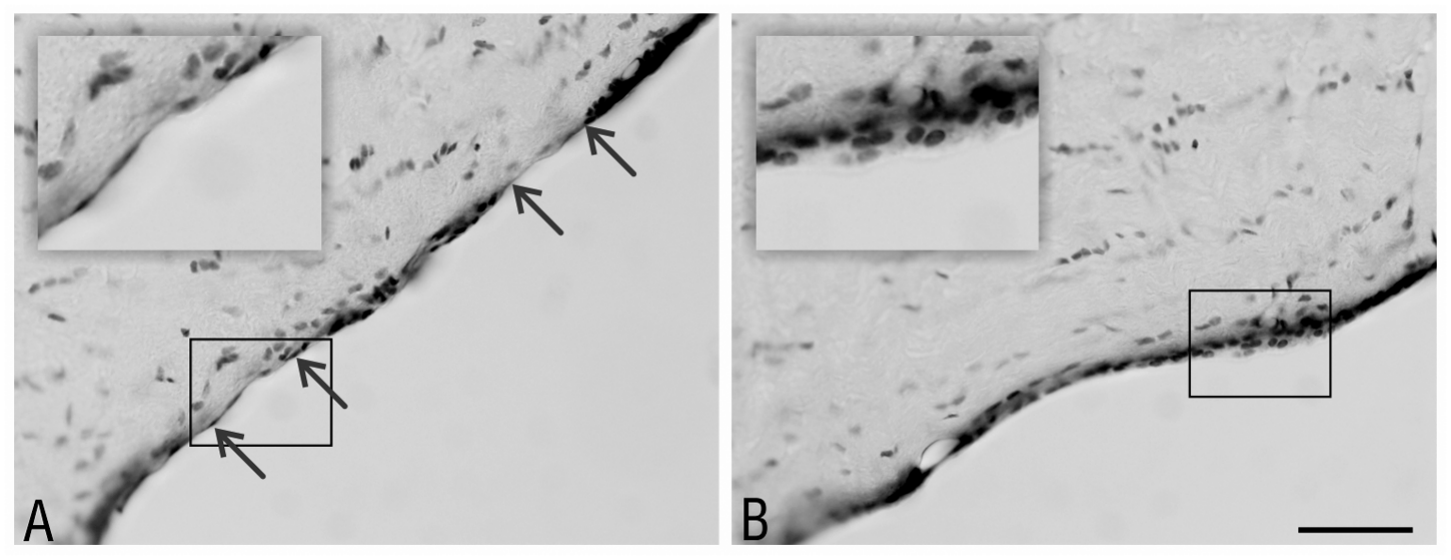
The characteristics of the ependymal discontinuity after epoxomicin administration. Microscopic view of the ependymal atrophy on the surface of the corpus callosum (A). (B) Normal control with an unchanged ventricular ependymal lining from the same section as (A), but from opposite side. The arrows indicate the borders between area of the ependymal atrophy and the existing normal ependymal lining. Sections of the rat brains at 8 weeks after epoxomicin administration are shown. Staining—Nissl method; scale bar = 50 μm.

Two weeks after the injection of DMSO, ependymal atrophy was only observed in 28% of the studied sections (Table 1). The frequency of ependymal atrophy observed after the injection of MG-132 and lactacystin was approximately 1.7-fold greater than that after DMSO application, but it was not significant. However, the frequency of ependymal atrophy observed in the sections from the epoxomicin-treated animals was significantly higher (p<0.001) than that after DMSO application (Table 1). Eight weeks after the injection of the studied substances, the areas of ependymal atrophy were localized on the surface of lateral septal nuclei and corpus callosum, close to the location of the ependymal discontinuity areas. Eight weeks after the injection of the DMSO and MG-132, the frequency of ependymal atrophy did not differ significantly. In the animals from the lactacystin group, the frequency of ependymal atrophy was significantly higher than that at two weeks after the application (p<0.05). At 8 weeks after the injection of lactacystin and epoxomicin, the frequency of ependymal atrophy present in the sections from those animals was significantly higher (p<0.001 and p<0.01, respectively) than those from animals treated with DMSO (Fig 5 and Table 1).

### Subependymal gliosis with glial scar and ependymal rosette formation

Ependymal denudation of the ventral wall of the injected lateral ventricle has caused direct contact between the striatum and lateral septal nucleus and produced an adhesion/glial scar between those structures. Compensating ventricular enlargement was usually present above the adhesion site. This caused further damage to the ependymal cells and led to specific repair processes, including the formation of subependymal glial nodules [24]. In contrast to a glial scar, which was often observed, glial nodules (Fig 4) were rarely present, with the exception of the epoxomicin-treated rats (Table 1). A glial scar, which partially closes the ventricular lumen, was mainly observed in the ventral part of the lateral ventricle in the areas of smooth ventricular surface rather than at the angles of the ventricles (Figs 1 and 2). At 2 weeks, the length of occlusion in the sagittal axis of the lateral ventricle was relatively small in the DMSO group (0.5 mm in average; Table 1). Eight weeks after DMSO treatment, the average length of the occlusion was 0.45 mm and was not significantly different from that of the earlier time point. The administration of MG-132, lactacystin or epoxomicin into the right lateral ventricle caused much more extensive changes within the ventricle and neighboring structures in all animals sacrificed 2 weeks and 8 weeks after the injection. Two weeks after the injection, MG-132 caused a 2-fold longer occlusion (p<0.05), while lactacystin and epoxomicin caused a 2.5-fold (p<0.01) longer occlusion compared to that observed in the control. At this time point, there were no significant differences in the length of the between the animals treated with the different proteasome inhibitors. Eight weeks after the injection, the occlusions were 2.5-fold longer in the MG-132 group (p<0.05), 3.4-fold longer in the lactacystin group (p<0.001) and 4-fold longer in the epoxomicin group (p<0.001) compared to the control group. Eight weeks after the injection, significant differences in the lengths of the occlusions were found between the animals treated with MG-132 and epoxomicin (p<0.05). The lengths of the occlusions between animals treated with the same chemical compound at 2 and 8 weeks after the injection were only significantly difference in the epoxomicin-treated rats (p<0.05). Table 1 presents the detailed data.

**Fig 6:**
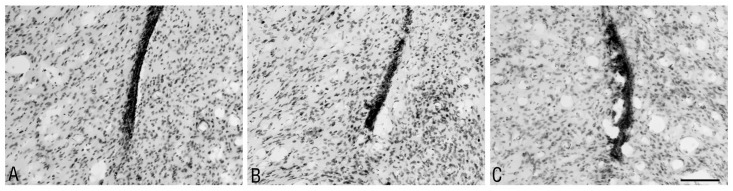
The morphology of the ependymal rosettes. Microscopic view of the ependymal rosettes formed at the ventral part of right lateral ventricle. Sections of the rat brains at 8 weeks after the administration of the studied substances are shown: A—DMSO, B—MG-132, and C—epoxomicin. Staining—Nissl method; scale bar = 100 μm.

On some of the sections, the adhesion was so prominent that it completely occluded the most ventral part of the lateral ventricle, sequestering the diverticuli of the surface ependyma to form ependymal rosettes (Fig 6).

Two weeks after the injection of the studied substances, ependymal rosettes were only observed in 16% of the sections from the MG-132-treated rats. However, eight weeks after the injection of the studied substances, ependymal rosettes were observed in all of the studied groups of animals (Table 1), most often in the sections from the MG-132- and epoxomicin-treated rats.

**Fig 7:**
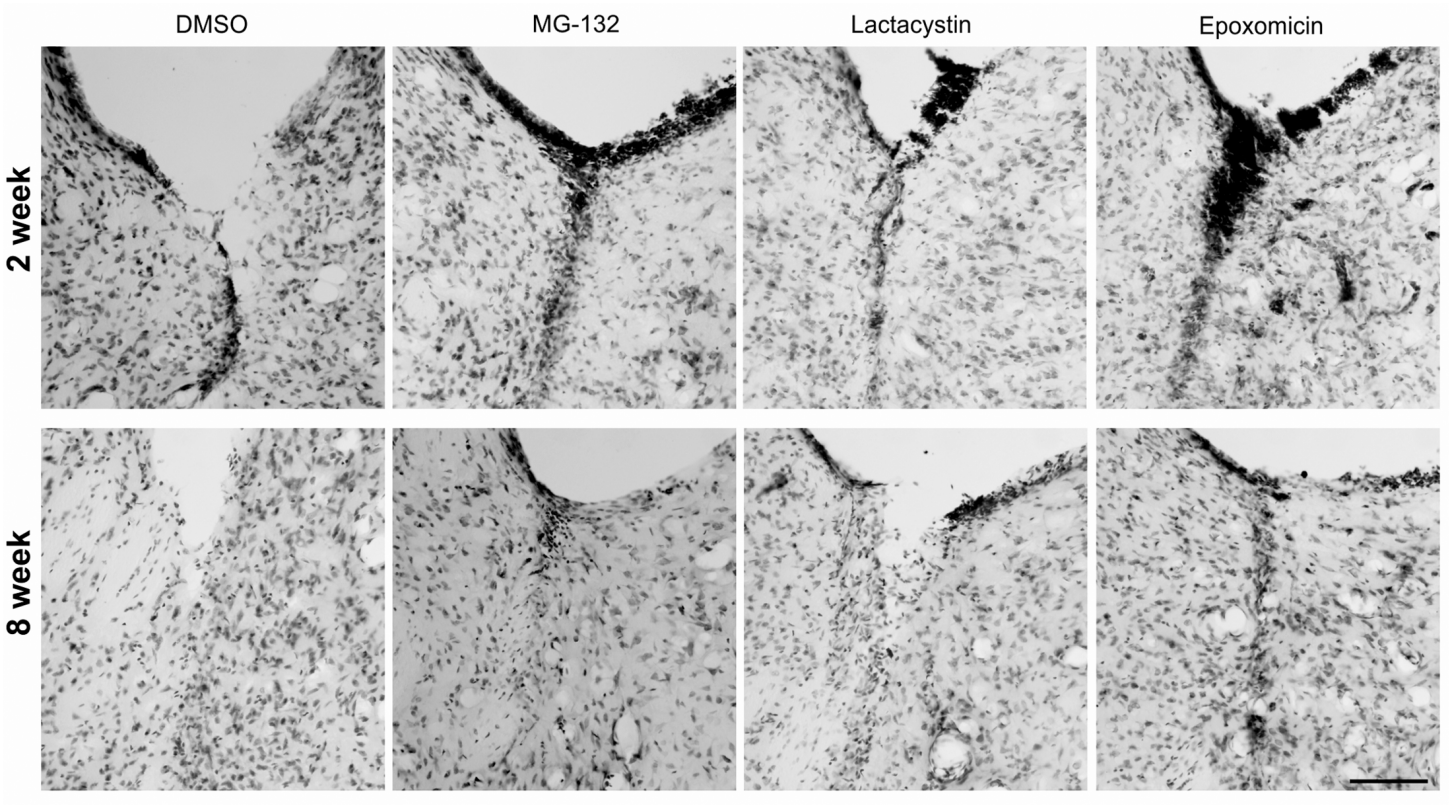
Evolution of the morphological changes within the rat lateral ventricle at 2 and 8 weeks after the administration of the studied substances. Microscopic view of the morphological changes (glial scar, mononuclear cells infiltration) associated with the adhesion area within lateral ventricle; a comparison of the view from coronal sections of the brains from the representative rats at 2 and 8 weeks after the administration of the studied substances– DMSO, MG-132, lactacystin and epoxomicin. Staining—Nissl method; scale bar = 100 μm.

Large numbers of glial cells with small, strong Nissl-stained nuclei were present in the proximity of the adhesion area (Fig 7).

**Fig 8:**
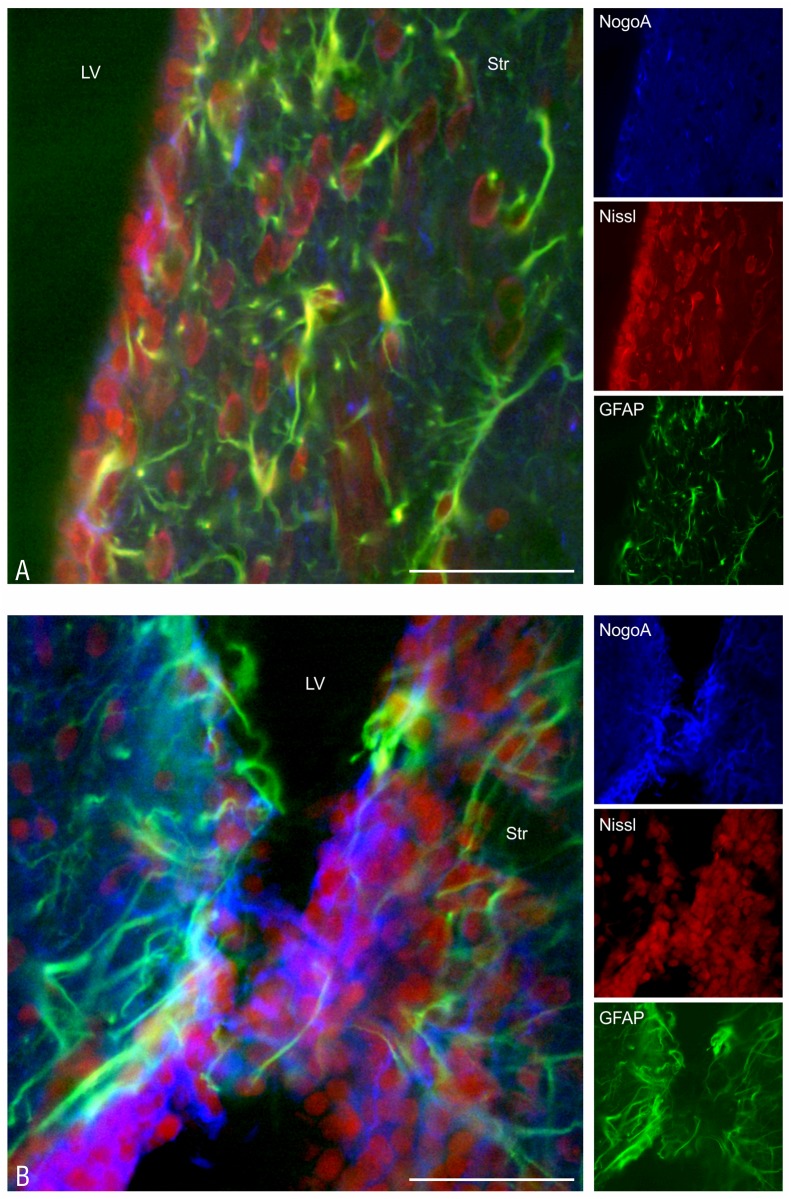
Glial scar formation in the lateral ventricle. Glial activation in the striatum (Str) and glial scar formation 2 weeks after the administration of DMSO (A) and epoxomicin (B) into the right lateral ventricle (LV). An anti-GFAP antibody was used as a marker of astroglia, an anti-NogoA antibody was used as a marker of oligodendroglia, and Neuro Trace Red stained the neuronal bodies and nuclei of both the vascular endothelial cells and glial cells. Scale bar—50 μm.

Combined histo- and immunofluorescence studies have shown an active process within the damaged wall of the lateral ventricle, in which GFAP-positive astrocytes are primarily involved (Fig 8). They are accompanied by a smaller number of NogoA-positive oligodendrocytes (Fig 8).

### Inflammatory cell infiltrations

**Fig 9:**
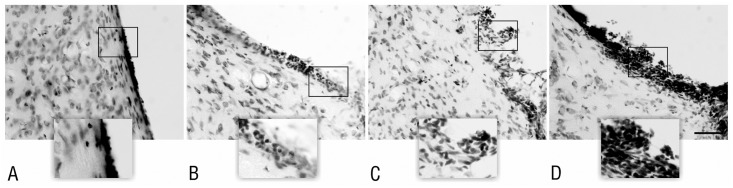
The characteristics of the mononuclear cell infiltrations after intraventricular proteasome inhibitor administration. Microscopic view of the mononuclear inflammatory cells observed at the lateral ventricular surface (A) and the different degrees of inflammatory cell infiltration: B—mild, C—moderate, and D—high. Sections of the rat brains at 2 weeks after the administration of the studied substances, A –DMSO, B, C—MG-132 and D—epoxomicin, are shown. Staining—Nissl method; scale bar = 50 μm.

Mononuclear cell infiltrations were observed in the areas where the ependyma was damaged (Fig 7). Mononuclear inflammatory cells were also observed at the ventricular surface and close to the ependymal debris, with various degrees of the subventricular neuropil inflammatory cell infiltrations (Fig 9).

Two weeks after the injection of DMSO, mononuclear cell infiltrations were observed in 44% of the studied sections. At the same time, but after the application of the proteasome inhibitors, the frequency of the mononuclear cell infiltrations was higher in the MG-132- and epoxomicin-treated rats (1.9-fold and 2.1-fold, respectively). The degree of mononuclear cell infiltration present on the sections (Table 1) was significantly more severe in the MG-132- (p = 0.015) and epoxomicin-treated (p<0.001) groups compared to the DMSO-treated group. At this time point, the degree of mononuclear cell infiltration in the sections from the lactacystin-treated rats did not differ significantly from that in the DMSO-treated rats (p = 0.114). Eight weeks after the injection of the studied substances, the frequency of the mononuclear cell infiltrations in the lactacystin- and epoxomicin-treated rats was higher (1.5-fold and 1.4-fold, respectively) than the DMSO-treated rats. The degree of mononuclear cell infiltration in the sections (Table 1) was significantly more severe in the lactacystin- (p = 0.006) and epoxomicin-treated (p = 0.002) groups compared to the DMSO-treated group. At this time point, the degree of the mononuclear cell infiltrations in the sections from the MG-132-treated rats were not significantly different from the DMSO-treated rats (p = 0.7).

**Fig 10:**
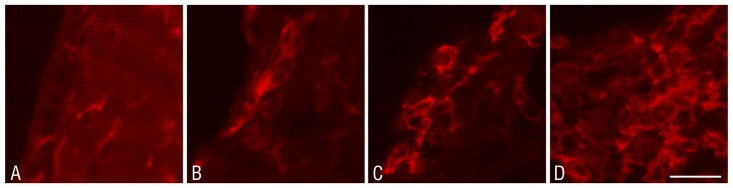
Microglia infiltrations within the walls of the lateral ventricle. The OX-42-positive cells (microglia) present within inflammatory cell infiltrations are shown in sections of the rat brains at 2 weeks after the administration of the studied substances: A—DMSO, B—MG-132, C—lactacystin and D—epoxomicin. Immunohistochemistry, scale bar = 50 μm.

The fact that the majority of glial nuclei visualized with Nissl staining were not colocalized with either the anti-GFAP or anti-NogoA staining suggests the involvement of the microglia in glial scar formation and inflammatory cell infiltration. The presence of the microglia within the inflammatory cell infiltrations has been confirmed by positive staining with an OX-42 antibody (Fig 10).

### Evidence of UPS impairment

**Fig 11:**
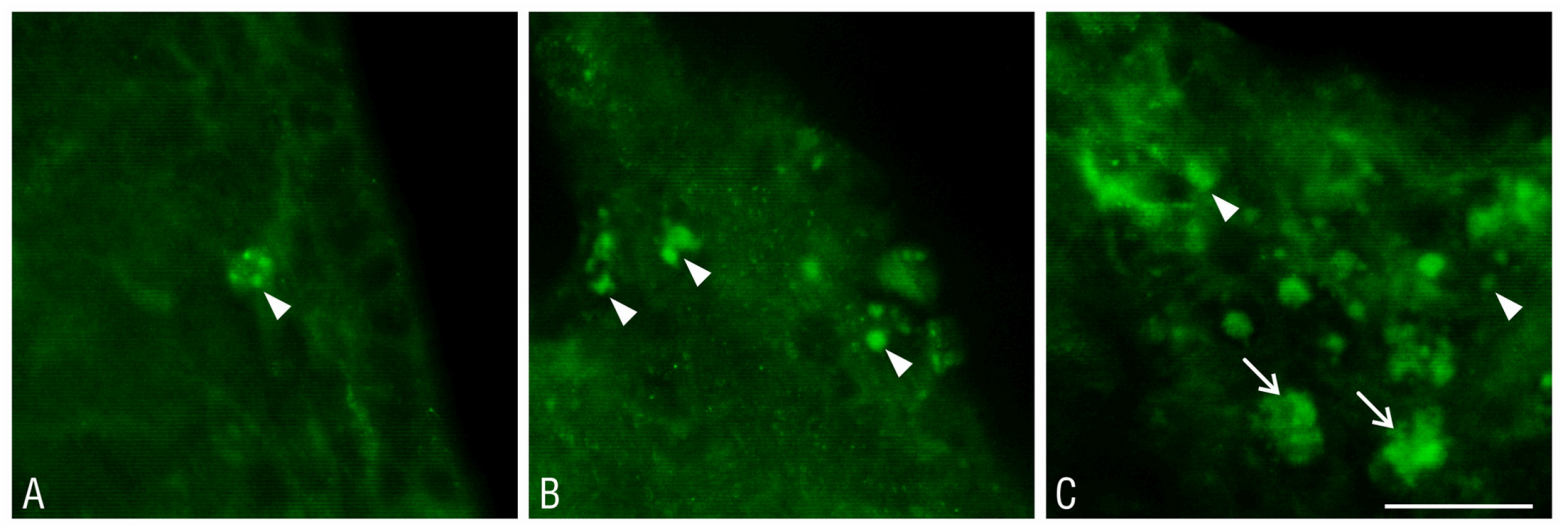
The morphology of the ubiquitin-positive aggregates in the lateral ventricular walls and subventricular area after the proteasome inhibitor treatment. The morphology of the ubiquitin-positive aggregates in the cells of the striatum at 2 weeks after the administration of the proteasome inhibitors into lateral ventricle is shown: **A**—single, small juxtanuclear aggregates (arrowhead), **B**—numerous larger cytoplasmic aggregates (arrowheads), and **C**—numerous cytoplasmic aggregates (arrowheads) and large clusters of ubiquitin-positive aggregates with a foamy morphology (arrows), which may provide the content of the phagocytic cells. Immunohistochemistry, scale bar = 50 μm.

In the studied material, ubiquitin-positive aggresome-like structures were observed in the cells within the structures adjacent to the lateral ventricle at 2 and 8 weeks after the administration of the proteasome inhibitors. Although ubiquitin-positive aggregates were observed after the administration of each tested proteasome inhibitor, the most marked changes were present in the striatum of the epoxomicin-treated animals. The ubiquitin-positive aggregates varied in terms of quantity, from single units to a great number of clusters, occupying almost the entire space of the cell (Fig 11), and in terms of morphology—from small juxtanuclear aggregates to larger cytoplasmic aggregates and large clusters of aggregates with a foamy morphology.

**Fig 12:**
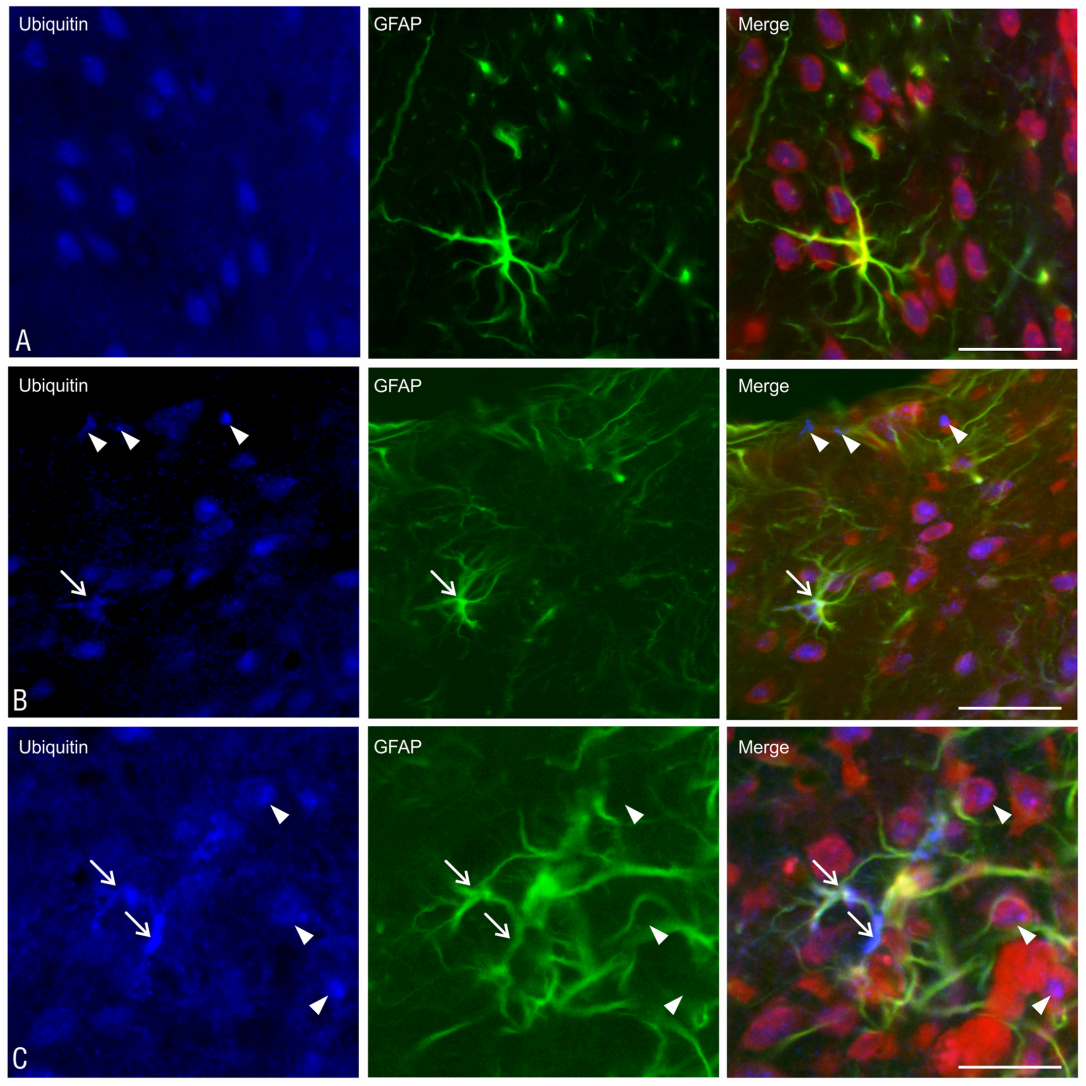
The ubiquitin-positive aggregates within the cells of subventricular area. The localization of the ubiquitin-positive aggregates in the cells of striatum after the administration of A—DMSO and B, C—epoxomicin is shown. The arrow points to the clusters of ubiquitin-positive aggregates in astrocytes, while the arrowheads indicate small ubiquitin inclusions in cells that are not astrocytes. Scale bars: A, B—50 μm; C—25 μm.

Ubiquitin-positive aggregates were observed in both the glial cells and in cells with a neuronal morphology (Fig 12). The area of their distribution corresponded to the presence of the glial scar area in the individual groups of animals. The highest density of cells containing ubiquitin-positive aggregates was localized to the injection site and progressively declined in sections placed anteriorly and posteriorly from the site of the proteasome inhibitor administration. In the control group, there were no such aggregates, and the distribution of the ubiquitin-positive components within the cells was relatively homogeneous (Fig 12).

## Discussion

Due to the therapeutic use of proteasome inhibitors, their influence on neural tissue has been analyzed for several years [4–9, 25], and one of the main conclusions is that their effect on the individual components of the nerve tissue—neurons and different populations of glial cells—varies [26, 27]. To date, the effect of proteasome inhibitors on the cells lining the ventricular system has been unexplored. This effect may be particularly important during the trials with intraventricular administration of the drugs. To the best of our knowledge, this is the first qualitative and quantitative description of how the experimental administration of different classes of proteasome inhibitors influences the ependymal cells.

Neurons, particularly dopaminergic neurons, seem to be very sensitive to the UPS alterations. Numerous in vitro [28, 29] and in vivo [9, 14, 25, 30] studies shown that the depletion of proteasome function leads to significant neurodegeneration and the formation of inclusion bodies.

Interestingly, various populations of glia in nervous tissue are differentially susceptible to proteasome inhibition. Astrocytes exhibit relatively low sensitivity to this type of inhibition and the pathological changes can be reversible [27, 31]. Extensive changes in the astrocytic cytoskeletal proteins, such as GFAP, nestin and vimentin were reported following proteasome inhibition, but the subsequent high expression of the heat shock proteins αB-crystallin and especially Hsp25 provided a protective effect for most (80%) of the studied cells [31]. Proteasome depletion in astrocytes also leads to an increased secretion of the antioxidant enzyme peroxiredoxin 6 (PRDX6) [32]. The changes in the astrocytes seem to be rather long lasting because it has also been reported that the astrocytes that survive one treatment with the proteasome inhibitors were protected against a second treatment by virtue of the antioxidant defenses [33].

In vitro studies have shown that oligodendrocytes seem to be much more sensitive to proteasome inhibition compared to astrocytes. The treatment of cultured primary oligodendrocytes with MG-132 caused mitochondrial dysfunction, oxidative stress, and, as a consequence, led to apoptotic cell death [34]. In contrast to the astrocytes, the disturbances of the cytoskeletal proteins in oligodendrocytes in response to proteasome inhibition induced massive apoptosis [31]. However, it must be mentioned that the results of other studies indicate that proteasome inhibition in oligodendrocytes may play a beneficial role by stabilizing the survival and differentiation signals [35, 36].

The reported influence of proteasome inhibition on microglia mainly focused on microglial activation and the accompanying neuronal degeneration [30, 36]. The results of more specific studies are more complex. The microglia showed a decreased survival rate and an increase in the pro-inflammatory response, including upregulation of NO and TNF-α secretion following proteasome inhibition with lactacystin [37], and a decrease in TNF-α secretion after MG-132 and ALLN (N-acetyl-Leu-Leu-Norleucinal) treatment [38]. In the present study, we showed that the degree of mononuclear cell infiltration is much more severe after the proteasome inhibitor treatment compared to that observed in the control group, and using the OX-42 antibody, we confirm that microglia are an important component of this infiltration. Further studies are needed to establish the microglial activation cascade.

The results of the study presented here clearly show, for the first time, that even a single application of the proteasome inhibitors MG-132, lactacystin or epoxomicin, which were dissolved in DMSO, into the lateral ventricle of adult rats led to an acute process of ependymal detachment from the walls of the lateral ventricle and ependymal cell death. Two weeks after the injection, the degree of ependymal discontinuity present in the sections was significantly more severe in the MG-132-, lactacystin- or epoxomicin-treated groups than that in the DMSO-treated group. It indicates that ependymal cells are highly sensitive to abnormal UPS function. Currently, it is unclear why these cells are so sensitive, and the molecular cascade of events triggered by the proteasome inhibitors leading to ependymal cell damage is unknown. The loss of ependymal cells and accompanying oedema observed in the adjacent nervous tissue was followed by the fusion of the striatum and lateral septum. This fusion, in turn, provoked further morphological changes within the wall of lateral ventricle that are related to the recruitment of different populations of glial cells and the formation of a glial scar. The observed chain of morphological events in the lateral ventricle after the administration of the proteasome inhibitors seems to be very similar to that observed after the administration of neuraminidase [39, 40], hydrogen peroxide [41], streptozotocin [42] and high doses of rhodamine dyes [43]. The changes were restricted to the lateral ventricle ipsilateral to the injection site, while sparing the contralateral ventricle, suggesting a diffusion-dependent toxicity mechanism.

The process of protein ubiquitination allows the subsequent transport of the given protein to the proteasomes and its proper degradation. The inhibition of the UPS leads to the intracellular accumulation of non-degraded, misfolded or unfolded ubiquitin-conjugated proteins, which are actively transported to the formations called aggresomes [44]. Aggresomes are cytoplasmic, juxtanuclear structures and are considered to be a cytoprotective response that serves to sequester potentially toxic, misfolded or unfolded, proteins and facilitate their clearance by autophagy [45]. As we have observed, the aggresome-like, ubiquitin-positive structures in the cells of the striatum and lateral septum indicate that the injected proteasome inhibitors must have reached not only the ependymal surface but also deeper into the basolateral region of the ependymal cells and subependymal zone. It is possible because the walls of the lateral ventricle are lined by ciliated, cuboidal ependymal cells, they are permeable and allow the passage of macromolecules through the intercellular spaces [24, 46].

In the present study, differences in the adhesion size were observed between the control groups and the MG-132-, lactacystin- and epoxomicin-treated groups. Two weeks after the injection, MG-132 caused a 2-fold longer occlusion, while lactacystin and epoxomicin cause a 2.5-fold longer occlusion compared to the control. Eight weeks after the injection, MG-132 caused a 2.5-fold longer occlusion, lactacystin caused a 3.4-fold longer occlusion and epoxomicin produced a 4-fold longer occlusion compared to the control. There were no significant differences in the length of the occlusion between the animals treated with different proteasome inhibitors at two weeks after the injection, but at 8 weeks after the injection, there were significant differences between animals treated with MG-132 and epoxomicin. Interestingly, epoxomicin was the only one of the studied proteasome inhibitors that caused significant changes in the occlusion length at 8 weeks after the administration compared to that observed after 2 weeks. The number of apoptotic bodies and the frequency of ependymal atrophy observed in the sections from the epoxomicin-treated animals were significantly higher at both 2 and 8 weeks after the administration than those after the DMSO application. The MG-132 treatment did not significantly influence those parameters, while lactacystin significantly influenced the frequency of ependymal atrophy at 8 weeks after the administration.

MG-132, which belongs to the group of peptide aldehydes, is a reversible proteasome inhibitor, while lactacystin and epoxomicin (belonging to β-lactone derivatives and epoxyketones, respectively) are irreversible inhibitors. Moreover, these inhibitors exert their effect upon different proteasome activities [1]. Primarily, epoxomicin potently inhibits the chymotrypsin-like catalytic activity of the proteasomes; however, the trypsin-like and the peptidyl-glutamyl peptide catalytic activities of the proteasomes are also inhibited by epoxomicin, but at 100- and 1,000-fold slower rates, respectively [47]. In contrast to peptide aldehyde proteasome inhibitors (e.g., MG-132), epoxomicin does not inhibit non-proteasomal proteases, such trypsin, chymotrypsin, papain, calpain, and cathepsin B. Lactacystin inhibits chymotrypsin- and trypsin-like catalytic activities, but epoxomicin is a more potent inhibitor of the chymotrypsin-like activity than lactacystin [47]. All of these differences may partially explain the observed phenomenon (the most powerful effect of epoxomicin).

A recent study also showed that nuclear factor κB (NF-κB) activation disturbs ependymal ciliogenesis and links astroglia and microglia activation and the subsequent neuroinflammation to hydrocephalus formation [48]. However, all three proteasome inhibitors used in the study, MG-132, lactacystin and epoxomicin, are known to decrease the activity of NF-κB in many populations of cells [47, 49]. Thus, it seems intriguing to determine whether they have the same influence on ependymal cells.

Another interesting finding of our study is that ependymal damage was also observed in the control groups (a single injection of 4 μl of DMSO). However, it was significantly less severe than that in the animals injected with the studied proteasome inhibitors. As an organic polar aprotic molecule, DMSO is commonly used as a solvent for the dissolution of small hydrophobic drug molecules [50]. Due to its ability to solubilize otherwise poorly soluble polar and nonpolar molecules it is utilized as the vehicle control-of-choice for many in vitro and in vivo studies (for review see [51]). In the previous studies, 100% DMSO vehicle has been shown to act as a neuroprotective agent in a rat model of traumatic brain injury [52]. Moreover, in additional studies, a 50% saline solution of DMSO was shown to have a neuroprotective effect in a rat model of cerebral ischemia [53]. On the other hand, some studies recognize that DMSO can be potentially toxic (e.g., by triggering apoptotic cell death), even in low doses such as 0.5% and 1.0% [51, 54–56]. Based on in vitro and in vivo studies, the mechanisms of DMSO-induced cell death include: plasma membrane pore formation, caspase-9 and caspase-3 activation, apoptosis-inducing factor translocation and poly-(ADP-ribose)-polymerase activation [51, 54, 56–58].

In the lateral ventricle, DMSO has been used in various concentrations for various purposes in several studies. A 1% solution of DMSO has been used as a solvent and was injected into the lateral ventricle in a study about the involvement of the heme oxygenase–carbon monoxide–cGMP pathway in nociception induced by an acute painful stimulus in rats [59]. A 3% solution of DMSO has been used in an in vivo study investigating the effect of chronic inhibition of brain phospholipase A_2_ in adult rats on the number of newborn mature neurons in the hippocampal dentate gyrus [60]. A higher dose of DMSO (40μl of 50% solution) was injected into the lateral ventricle in a study testing the role of ERK activation in the neuroprotective mechanisms of hypothermia after cardiac arrest [61]. The results of the behavioural tests performed after the intracerebroventricular chronic administration of DMSO in rats (single injections of 2.5, 5 and 10% solutions in 2 μl of saline per day for two weeks) suggest that DMSO may be appropriate for use as an adjuvant therapy for the prevention of memory impairments in experimental models of Alzheimer’s disease [62]. None of the aforementioned studies presented information about the changes in the morphology of the lateral ventricle. Moreover, none of them showed a detailed morphological analysis, and some of them [59, 62] only discussed the results of the behavioural tests. Our data provide new valuable information indicating that DMSO can be potentially toxic to ependymal cells, and even a single injection may lead to changes in the ventricular system, causing possible disturbances in the CSF flow.

Intraventricular hemorrhage is an additional factor that may have a potential influence on the morphological changes observed in the lateral ventricle in this study. Although no evidence of intraventricular haemorrhage was observed in our studies at 2 and 8 weeks after the surgery, we cannot rule out the possibility that during the early postoperative period some micro hemorrhages were present. Previous studies demonstrated that the thrombin and iron in the blood caused ventricular wall damage, periventricular blood-brain barrier disruption, and, subsequently, significant hydrocephalus [63, 64]. Despite this, the significant differences between the studied groups described in the present study support the theory that proteasome inhibition is an important contributing factor to the occlusion of the lateral ventricle.

## Conclusions

The administration of chemical reagents (e.g., DMSO, proteasome inhibitors) to the lateral ventricle of the rat leads to ependymal cell damage. Regardless of the type of damage (mechanical, chemical), it induces cellular, mostly glial, activation, leading to the formation of glial scars, a reduction in the volume of the lateral ventricle, and most likely, perturbations in the flow of the cerebrospinal fluid. The size of the formed glial scar is significantly larger after the administration of proteasome inhibitors, suggesting that degenerative/repair processes are more severe. A different pattern of morphological changes was observed after the administration of epoxomicin, which supports the findings of previous reports about the heterogeneity of the activity of different classes of proteasome inhibitors.

There were no significant differences in the length of the glial scar, as measured in the sagittal axis, between the second and eighth weeks after intraventricular administration of DMSO and all proteasome inhibitors, with the exception of epoxomicin, which may suggest that the major repair processes occur during the first two weeks after injury. However, the evidence of secondary lesions during the six weeks between two experimental endpoints, namely the formation of ependymal rosettes and the increase frequency of ependymal discontinuity at the surface of the corpus callosum, indicates the sustainability of the ongoing repair processes.

Intraventricular administration of proteasome inhibitors causes a change in the morphology of the intracellular ubiquitin-positive components. The most substantial changes were observed in the striatum of the epoxomicin-treated animals and milder –changes were observed after lactacystin and MG-132 treatment. Morphologically, the ubiquitin-positive aggregates corresponded to aggresomes, indicating impaired activity of the ubiquitin-proteasome system. The distribution of the aggresome-like structures coincided with the occurrence of glial scars in the given groups of animals treated with proteasome inhibitors. Structures with an aggresome-like morphology were not observed in the control group.

Our results indicate that all studied classes of proteasome inhibitors are detrimental to the ependymal cells, but to different extents, and may cause severe changes in the ventricular system. The safety of their use in therapeutic strategies to attenuate intracerebral hemorrhagic injury and in treating brain cancer will require further studies.

## Supporting Information

S1 File Supplementary data. (DOCX)
