# Supplementary material for: Morphological Changes within the Rat Lateral Ventricle after the Administration of Proteasome Inhibitors
Source: PLoS One. 2015 Oct 19;10(10):e0140536. doi: 10.1371/journal.pone.0140536 (PMC4610704; doi:10.1371/journal.pone.0140536)
Supplement: S1 File — (DOCX) [file pone.0140536.s001.docx]

Supplementary data for **Morphological changes within the rat lateral ventricle after the administration of proteasome inhibitors**

To estimate the extent of adhesion between the striatum and septum (glial scar) in each rat, all Nissl-stained serial coronal sections were compared to the atlas images [[1](#_ENREF_1)], and the distance (in mm) from bregma point in the sagittal axis was calculated. After determining the length of the adhesion and establishing that the adhesion can reach a maximum in a section located 1.6 mm anteriorly and 0.8 mm posteriorly from the bregma point, the major morphological alterations in the tissue were quantitatively analyzed by light microscopy at 10x and 40x magnifications. In the first step, five systematically and randomly chosen coronal sections of the brain were taken from 1.7 mm anterior to 1.0 mm posterior from the bregma point from each animal [[1](#_ENREF_1)]. On those sections, the following parameters were estimated within the wall of the lateral ventricle and subventricular area: the presence and localisation of ependymal discontinuity, the presence and number of apoptotic bodies per section, the presence of ependymal atrophy, the presence of a glial scar, the presence of ependymal rosettes, the presence of glial nodules, and the presence of mononuclear inflammatory cell infiltrations. Additional scoring of the ependymal discontinuity and mononuclear inflammatory cell infiltrations observed in the rat brain coronal sections was performed. They were scored as follows: none, mild, moderate, and high degree. In the table below the raw data obtained during evaluation are present.

| week | group | rat | n | Atrophy | Apoptosis | Apoptosis [cells/section] | Ependymal denudation | Ependymal denudation [grade] | ED- striatum | ED-septum | ED-CC | Rosette | Glial scar | Glial nodules | Glial nodules [per section] | mononuclear inflamatory cells | mononuclear inflamatory cells [grade] |
| --- | --- | --- | --- | --- | --- | --- | --- | --- | --- | --- | --- | --- | --- | --- | --- | --- | --- |
| 2 | DMSO | Ra 11-70 | 1 | 0 | 1 | 3 | 0 | 0 | 0 | 0 | 0 | 0 | 0 | 0 | 0 | 0 | 0 |
| 2 | DMSO | Ra 11-70 | 1 | 0 | 1 | 7 | 0 | 0 | 0 | 0 | 0 | 0 | 0 | 1 | 2 | 1 | 2 |
| 2 | DMSO | Ra 11-70 | 1 | 1 | 1 | 2 | 1 | 1 | 0 | 0 | 1 | 0 | 0 | 1 | 4 | 1 | 2 |
| 2 | DMSO | Ra 11-70 | 1 | 0 | 1 | 1 | 1 | 1 | 0 | 1 | 1 | 0 | 0 | 0 | 0 | 1 | 3 |
| 2 | DMSO | Ra 11-70 | 1 | 0 | 0 | 0 | 1 | 2 | 0 | 1 | 1 | 0 | 0 | 0 | 0 | 1 | 2 |
| 2 | DMSO | Ra 11-71 | 1 | 0 | 0 | 0 | 0 | 0 | 0 | 0 | 0 | 0 | 0 | 0 | 0 | 0 | 0 |
| 2 | DMSO | Ra 11-71 | 1 | 0 | 0 | 0 | 0 | 0 | 0 | 0 | 0 | 0 | 0 | 0 | 0 | 0 | 0 |
| 2 | DMSO | Ra 11-71 | 1 | 0 | 0 | 0 | 0 | 0 | 0 | 0 | 0 | 0 | 0 | 0 | 0 | 0 | 0 |
| 2 | DMSO | Ra 11-71 | 1 | 0 | 1 | 3 | 1 | 2 | 1 | 1 | 0 | 0 | 1 | 1 | 2 | 1 | 2 |
| 2 | DMSO | Ra 11-71 | 1 | 0 | 0 | 0 | 1 | 1 | 0 | 1 | 0 | 0 | 0 | 0 | 0 | 1 | 3 |
| 2 | DMSO | Ra 11-72 | 1 | 0 | 0 | 0 | 0 | 0 | 0 | 0 | 0 | 0 | 0 | 0 | 0 | 0 | 0 |
| 2 | DMSO | Ra 11-72 | 1 | 0 | 1 | 2 | 0 | 0 | 0 | 0 | 0 | 0 | 0 | 0 | 0 | 0 | 0 |
| 2 | DMSO | Ra 11-72 | 1 | 1 | 1 | 4 | 0 | 0 | 0 | 0 | 0 | 0 | 1 | 0 | 0 | 1 | 1 |
| 2 | DMSO | Ra 11-72 | 1 | 0 | 1 | 2 | 0 | 0 | 0 | 0 | 0 | 0 | 0 | 0 | 0 | 0 | 0 |
| 2 | DMSO | Ra 11-72 | 1 | 1 | 1 | 1 | 1 | 1 | 0 | 1 | 0 | 0 | 0 | 0 | 0 | 0 | 0 |
| 2 | Epox | Ra 11-85 | 1 | 1 | 0 | 0 | 1 | 1 | 0 | 1 | 0 | 0 | 1 | 1 | 1 | 1 | 3 |
| 2 | Epox | Ra 11-85 | 1 | 1 | 1 | 6 | 1 | 3 | 1 | 1 | 0 | 0 | 1 | 1 | 1 | 1 | 3 |
| 2 | Epox | Ra 11-85 | 1 | 1 | 1 | 10 | 1 | 3 | 1 | 1 | 0 | 0 | 1 | 0 | 0 | 1 | 3 |
| 2 | Epox | Ra 11-85 | 1 | 1 | 1 | 2 | 1 | 2 | 0 | 1 | 0 | 0 | 0 | 0 | 0 | 1 | 2 |
| 2 | Epox | Ra 11-85 | 1 | 1 | 1 | 2 | 1 | 2 | 0 | 1 | 1 | 0 | 0 | 0 | 0 | 1 | 2 |
| 2 | Epox | Ra 11-86 | 1 | 0 | 1 | 2 | 0 | 0 | 0 | 0 | 0 | 0 | 0 | 0 | 0 | 0 | 0 |
| 2 | Epox | Ra 11-86 | 1 | 1 | 1 | 7 | 1 | 3 | 1 | 1 | 0 | 0 | 1 | 0 | 0 | 1 | 3 |
| 2 | Epox | Ra 11-86 | 1 | 1 | 1 | 5 | 1 | 3 | 1 | 1 | 0 | 0 | 1 | 1 | 1 | 1 | 3 |
| 2 | Epox | Ra 11-86 | 1 | 1 | 1 | 11 | 1 | 3 | 0 | 1 | 0 | 0 | 1 | 1 | 1 | 1 | 3 |
| 2 | Epox | Ra 11-86 | 1 | 1 | 1 | 7 | 1 | 3 | 0 | 1 | 1 | 0 | 1 | 0 | 0 | 1 | 3 |
| 2 | Epox | Ra 11-87 | 1 | 0 | 1 | 4 | 1 | 3 | 1 | 1 | 0 | 0 | 1 | 1 | 1 | 1 | 2 |
| 2 | Epox | Ra 11-87 | 1 | 1 | 1 | 6 | 1 | 3 | 0 | 1 | 0 | 0 | 1 | 0 | 0 | 1 | 2 |
| 2 | Epox | Ra 11-87 | 1 | 1 | 0 | 0 | 1 | 3 | 0 | 1 | 0 | 0 | 1 | 1 | 1 | 1 | 3 |
| 2 | Epox | Ra 11-87 | 1 | 1 | 0 | 0 | 1 | 2 | 0 | 1 | 0 | 0 | 0 | 0 | 0 | 1 | 3 |
| 2 | Epox | Ra 11-87 | 1 | 1 | 1 | 5 | 1 | 2 | 0 | 1 | 1 | 0 | 0 | 0 | 0 | 1 | 2 |
| 2 | MG-132 | Ra 11-75 | 1 | 0 | 1 | 6 | 1 | 2 | 1 | 1 | 0 | 1 | 1 | 0 | 0 | 1 | 2 |
| 2 | MG-132 | Ra 11-75 | 1 | 0 | 1 | 6 | 1 | 1 | 0 | 1 | 0 | 1 | 1 | 1 | 1 | 1 | 2 |
| 2 | MG-132 | Ra 11-75 | 1 | 0 | 1 | 3 | 1 | 1 | 0 | 1 | 0 | 1 | 1 | 0 | 0 | 1 | 1 |
| 2 | MG-132 | Ra 11-75 | 1 | 1 | 1 | 2 | 1 | 3 | 1 | 1 | 0 | 0 | 0 | 0 | 0 | 1 | 3 |
| 2 | MG-132 | Ra 11-75 | 1 | 1 | 1 | 4 | 1 | 2 | 1 | 1 | 0 | 0 | 0 | 0 | 0 | 1 | 2 |
| 2 | MG-132 | Ra 11-76 | 1 | 0 | 1 | 1 | 0 | 0 | 0 | 0 | 0 | 0 | 0 | 0 | 0 | 0 | 0 |
| 2 | MG-132 | Ra 11-76 | 1 | 0 | 1 | 4 | 1 | 1 | 0 | 1 | 0 | 0 | 0 | 0 | 0 | 0 | 0 |
| 2 | MG-132 | Ra 11-76 | 1 | 1 | 1 | 1 | 1 | 2 | 1 | 1 | 0 | 0 | 1 | 1 | 1 | 1 | 3 |
| 2 | MG-132 | Ra 11-76 | 1 | 1 | 1 | 7 | 1 | 3 | 1 | 1 | 0 | 0 | 1 | 1 | 1 | 1 | 3 |
| 2 | MG-132 | Ra 11-76 | 1 | 1 | 1 | 2 | 1 | 2 | 0 | 1 | 1 | 0 | 0 | 0 | 0 | 1 | 2 |
| 2 | MG-132 | Ra 11-77 | 1 | 1 | 1 | 3 | 1 | 2 | 1 | 1 | 0 | 1 | 1 | 0 | 0 | 1 | 2 |
| 2 | MG-132 | Ra 11-77 | 1 | 0 | 1 | 5 | 1 | 2 | 1 | 1 | 0 | 1 | 1 | 0 | 0 | 1 | 2 |
| 2 | MG-132 | Ra 11-77 | 1 | 1 | 1 | 4 | 1 | 3 | 0 | 1 | 0 | 1 | 1 | 0 | 0 | 1 | 3 |
| 2 | MG-132 | Ra 11-77 | 1 | 1 | 1 | 3 | 1 | 2 | 0 | 1 | 0 | 1 | 1 | 1 | 2 | 1 | 2 |
| 2 | MG-132 | Ra 11-77 | 1 | 0 | 1 | 2 | 1 | 1 | 0 | 1 | 0 | 0 | 0 | 1 | 1 | 1 | 2 |
| 2 | Lac | Ra 11-81 | 1 | 0 | 0 | 0 | 0 | 0 | 0 | 0 | 0 | 0 | 0 | 1 | 1 | 0 | 0 |
| 2 | Lac | Ra 11-81 | 1 | 0 | 1 | 2 | 1 | 1 | 0 | 1 | 0 | 0 | 1 | 0 | 0 | 1 | 2 |
| 2 | Lac | Ra 11-81 | 1 | 1 | 1 | 6 | 1 | 1 | 1 | 1 | 0 | 0 | 1 | 0 | 0 | 1 | 2 |
| 2 | Lac | Ra 11-81 | 1 | 1 | 1 | 4 | 1 | 1 | 0 | 1 | 0 | 0 | 1 | 1 | 1 | 1 | 3 |
| 2 | Lac | Ra 11-81 | 1 | 0 | 1 | 3 | 1 | 1 | 1 | 0 | 0 | 0 | 0 | 0 | 0 | 1 | 1 |
| 2 | Lac | Ra 11-83 | 1 | 0 | 0 | 0 | 0 | 0 | 0 | 0 | 0 | 0 | 0 | 0 | 0 | 0 | 0 |
| 2 | Lac | Ra 11-83 | 1 | 1 | 1 | 5 | 1 | 2 | 1 | 1 | 0 | 0 | 0 | 0 | 0 | 0 | 0 |
| 2 | Lac | Ra 11-83 | 1 | 0 | 1 | 4 | 1 | 1 | 1 | 1 | 0 | 0 | 1 | 0 | 0 | 1 | 2 |
| 2 | Lac | Ra 11-83 | 1 | 0 | 1 | 3 | 1 | 2 | 0 | 1 | 0 | 0 | 1 | 0 | 0 | 1 | 3 |
| 2 | Lac | Ra 11-83 | 1 | 1 | 1 | 4 | 1 | 3 | 1 | 1 | 0 | 0 | 0 | 0 | 0 | 1 | 3 |
| 2 | Lac | Ra 11-84 | 1 | 0 | 1 | 3 | 0 | 0 | 0 | 0 | 0 | 0 | 0 | 0 | 0 | 0 | 0 |
| 2 | Lac | Ra 11-84 | 1 | 0 | 1 | 5 | 1 | 1 | 0 | 1 | 0 | 0 | 0 | 0 | 0 | 1 | 1 |
| 2 | Lac | Ra 11-84 | 1 | 1 | 1 | 4 | 1 | 1 | 0 | 1 | 0 | 0 | 1 | 0 | 0 | 1 | 2 |
| 2 | Lac | Ra 11-84 | 1 | 1 | 1 | 4 | 1 | 3 | 0 | 1 | 0 | 0 | 1 | 1 | 1 | 1 | 3 |
| 2 | Lac | Ra 11-84 | 1 | 1 | 1 | 6 | 1 | 2 | 0 | 1 | 1 | 0 | 0 | 0 | 0 | 1 | 3 |
| 8 | DMSO | Ra 09-03 | 1 | 0 | 0 | 0 | 0 | 0 | 0 | 0 | 0 | 0 | 0 | 0 | 0 | 0 | 0 |
| 8 | DMSO | Ra 09-03 | 1 | 1 | 1 | 8 | 1 | 3 | 0 | 1 | 1 | 0 | 1 | 0 | 0 | 1 | 2 |
| 8 | DMSO | Ra 09-03 | 1 | 1 | 1 | 12 | 1 | 3 | 1 | 1 | 1 | 0 | 1 | 1 | 1 | 1 | 2 |
| 8 | DMSO | Ra 09-03 | 1 | 0 | 1 | 5 | 1 | 2 | 0 | 1 | 1 | 0 | 0 | 0 | 0 | 1 | 1 |
| 8 | DMSO | Ra 09-03 | 1 | 0 | 1 | 4 | 1 | 1 | 0 | 0 | 1 | 0 | 0 | 0 | 0 | 0 | 0 |
| 8 | DMSO | Ra 09-04 | 1 | 0 | 0 | 0 | 0 | 0 | 0 | 0 | 0 | 0 | 0 | 0 | 0 | 0 | 0 |
| 8 | DMSO | Ra 09-04 | 1 | 1 | 1 | 5 | 1 | 3 | 1 | 1 | 0 | 1 | 1 | 1 | 2 | 1 | 1 |
| 8 | DMSO | Ra 09-04 | 1 | 1 | 1 | 4 | 1 | 2 | 0 | 1 | 1 | 0 | 1 | 0 | 0 | 1 | 3 |
| 8 | DMSO | Ra 09-04 | 1 | 1 | 0 | 0 | 1 | 2 | 0 | 1 | 1 | 0 | 0 | 1 | 1 | 1 | 1 |
| 8 | DMSO | Ra 09-04 | 1 | 1 | 1 | 2 | 1 | 2 | 0 | 1 | 1 | 0 | 0 | 0 | 1 | 0 | 0 |
| 8 | DMSO | Ra 09-05 | 1 | 0 | 0 | 0 | 0 | 0 | 0 | 0 | 0 | 0 | 0 | 0 | 0 | 0 | 0 |
| 8 | DMSO | Ra 09-05 | 1 | 1 | 1 | 3 | 1 | 1 | 0 | 1 | 0 | 1 | 1 | 0 | 0 | 1 | 1 |
| 8 | DMSO | Ra 09-05 | 1 | 1 | 1 | 5 | 1 | 2 | 1 | 1 | 1 | 1 | 1 | 0 | 0 | 1 | 1 |
| 8 | DMSO | Ra 09-05 | 1 | 1 | 1 | 11 | 1 | 2 | 1 | 1 | 1 | 0 | 1 | 0 | 0 | 1 | 2 |
| 8 | DMSO | Ra 09-05 | 1 | 1 | 1 | 3 | 1 | 2 | 1 | 1 | 1 | 0 | 0 | 0 | 0 | 1 | 1 |
| 8 | DMSO | Ra 10-36 | 1 | 0 | 0 | 0 | 0 | 0 | 0 | 0 | 0 | 0 | 0 | 0 | 0 | 0 | 0 |
| 8 | DMSO | Ra 10-36 | 1 | 0 | 1 | 2 | 0 | 0 | 0 | 0 | 0 | 0 | 0 | 0 | 0 | 0 | 0 |
| 8 | DMSO | Ra 10-36 | 1 | 1 | 1 | 2 | 1 | 2 | 0 | 1 | 1 | 0 | 0 | 0 | 0 | 1 | 1 |
| 8 | DMSO | Ra 10-36 | 1 | 0 | 1 | 2 | 0 | 0 | 0 | 0 | 0 | 0 | 0 | 0 | 0 | 1 | 2 |
| 8 | DMSO | Ra 10-36 | 1 | 0 | 1 | 1 | 1 | 1 | 0 | 1 | 0 | 0 | 0 | 0 | 0 | 0 | 0 |
| 8 | DMSO | Ra 10-37 | 1 | 0 | 0 | 0 | 0 | 0 | 0 | 0 | 0 | 0 | 0 | 0 | 0 | 0 | 0 |
| 8 | DMSO | Ra 10-37 | 1 | 1 | 1 | 5 | 1 | 1 | 1 | 1 | 0 | 0 | 1 | 0 | 0 | 1 | 2 |
| 8 | DMSO | Ra 10-37 | 1 | 0 | 1 | 3 | 1 | 1 | 0 | 1 | 0 | 0 | 1 | 0 | 0 | 1 | 2 |
| 8 | DMSO | Ra 10-37 | 1 | 0 | 1 | 6 | 1 | 1 | 0 | 1 | 0 | 1 | 1 | 0 | 0 | 1 | 2 |
| 8 | DMSO | Ra 10-37 | 1 | 1 | 1 | 4 | 0 | 0 | 0 | 0 | 0 | 0 | 0 | 1 | 1 | 1 | 1 |
| 8 | Epox | Ra 09-12 | 1 | 0 | 1 | 1 | 0 | 0 | 0 | 0 | 0 | 0 | 0 | 0 | 0 | 1 | 1 |
| 8 | Epox | Ra 09-12 | 1 | 0 | 1 | 10 | 1 | 3 | 1 | 1 | 1 | 0 | 1 | 0 | 0 | 1 | 3 |
| 8 | Epox | Ra 09-12 | 1 | 0 | 1 | 7 | 1 | 3 | 0 | 1 | 0 | 0 | 1 | 0 | 0 | 1 | 3 |
| 8 | Epox | Ra 09-12 | 1 | 1 | 1 | 7 | 1 | 3 | 0 | 1 | 0 | 0 | 1 | 0 | 0 | 1 | 3 |
| 8 | Epox | Ra 09-12 | 1 | 1 | 1 | 8 | 1 | 3 | 0 | 1 | 1 | 0 | 0 | 0 | 0 | 1 | 2 |
| 8 | Epox | Ra 09-13 | 1 | 0 | 1 | 3 | 0 | 0 | 0 | 0 | 0 | 0 | 0 | 0 | 0 | 0 | 0 |
| 8 | Epox | Ra 09-13 | 1 | 0 | 1 | 4 | 0 | 0 | 0 | 0 | 0 | 0 | 0 | 0 | 0 | 0 | 0 |
| 8 | Epox | Ra 09-13 | 1 | 1 | 1 | 12 | 1 | 2 | 0 | 1 | 0 | 0 | 1 | 0 |  | 1 | 3 |
| 8 | Epox | Ra 09-13 | 1 | 1 | 1 | 11 | 1 | 2 | 0 | 1 | 0 | 0 | 1 | 0 |  | 1 | 3 |
| 8 | Epox | Ra 09-13 | 1 | 0 | 1 | 3 | 1 | 1 | 0 | 1 | 1 | 0 | 0 | 1 | 2 | 1 | 2 |
| 8 | Epox | Ra 09-14 | 1 | 0 | 1 | 6 | 1 | 1 | 1 | 1 | 0 | 1 | 1 | 0 | 0 | 1 | 2 |
| 8 | Epox | Ra 09-14 | 1 | 1 | 1 | 21 | 1 | 2 | 1 | 1 | 1 | 1 | 1 | 0 | 0 | 1 | 2 |
| 8 | Epox | Ra 09-14 | 1 | 1 | 1 | 9 | 1 | 2 | 1 | 1 | 0 | 0 | 1 | 0 | 0 | 1 | 3 |
| 8 | Epox | Ra 09-14 | 1 | 1 | 1 | 5 | 1 | 3 | 0 | 1 | 1 | 0 | 0 | 0 | 0 | 1 | 3 |
| 8 | Epox | Ra 09-14 | 1 | 1 | 0 | 0 | 1 | 3 | 0 | 1 | 1 |  | 0 | 1 | 1 | 1 | 2 |
| 8 | Epox | Ra 10-58 | 1 | 0 | 1 | 2 | 1 | 1 | 1 | 1 | 0 | 1 | 1 | 0 | 0 | 1 | 2 |
| 8 | Epox | Ra 10-58 | 1 | 1 | 1 | 11 | 1 | 3 | 1 | 1 | 1 | 1 | 1 | 1 | 2 | 1 | 3 |
| 8 | Epox | Ra 10-58 | 1 | 1 | 1 | 7 | 1 | 3 | 0 | 1 | 1 | 1 | 1 | 0 | 1 | 1 | 3 |
| 8 | Epox | Ra 10-58 | 1 | 1 | 1 | 9 | 1 | 2 | 0 | 1 | 1 | 1 | 1 | 1 | 1 | 1 | 2 |
| 8 | Epox | Ra 10-58 | 1 | 1 | 1 | 12 | 1 | 2 | 0 | 1 | 1 | 0 | 1 | 1 | 2 | 1 | 2 |
| 8 | Epox | Ra 10-56 | 1 | 0 | 1 | 2 | 0 | 0 | 0 | 0 | 0 | 0 | 0 | 0 | 0 | 0 | 0 |
| 8 | Epox | Ra 10-56 | 1 | 1 | 1 | 5 | 0 | 0 | 0 | 0 | 0 | 0 | 0 | 0 | 0 | 1 | 1 |
| 8 | Epox | Ra 10-56 | 1 | 1 | 1 | 3 | 1 | 1 | 0 | 1 | 0 | 0 | 1 | 0 | 0 | 1 | 2 |
| 8 | Epox | Ra 10-56 | 1 | 1 | 1 | 4 | 1 | 3 | 1 | 1 | 0 | 0 | 1 | 0 | 0 | 1 | 3 |
| 8 | Epox | Ra 10-56 | 1 | 1 | 1 | 10 | 1 | 2 | 0 | 1 | 0 | 0 | 0 | 0 | 0 | 1 | 2 |
| 8 | MG-132 | Ra 09-06 | 1 | 0 | 1 | 3 | 0 | 0 | 0 | 0 | 0 | 0 | 0 | 0 | 0 | 0 | 0 |
| 8 | MG-132 | Ra 09-06 | 1 | 0 | 1 | 5 | 0 | 0 | 0 | 0 | 0 | 0 | 1 | 0 | 0 | 0 | 0 |
| 8 | MG-132 | Ra 09-06 | 1 | 1 | 1 | 4 | 1 | 2 | 0 | 1 | 0 | 0 | 1 | 0 | 0 | 1 | 2 |
| 8 | MG-132 | Ra 09-06 | 1 | 0 | 1 | 5 | 1 | 1 | 0 | 1 | 1 | 0 | 0 | 0 | 0 | 1 | 1 |
| 8 | MG-132 | Ra 09-06 | 1 | 0 | 1 | 2 | 1 | 1 | 0 | 1 | 1 | 0 | 0 | 1 | 1 | 0 | 0 |
| 8 | MG-132 | Ra 09-07 | 1 | 0 | 1 | 4 | 0 | 0 | 0 | 0 | 0 | 0 | 0 | 0 | 0 | 0 | 0 |
| 8 | MG-132 | Ra 09-07 | 1 | 1 | 1 | 18 | 1 | 3 | 1 | 1 | 1 | 1 | 1 | 0 | 0 | 1 | 3 |
| 8 | MG-132 | Ra 09-07 | 1 | 1 | 1 | 14 | 1 | 3 | 1 | 1 | 1 | 1 | 1 | 0 | 0 | 1 | 2 |
| 8 | MG-132 | Ra 09-07 | 1 | 0 | 1 | 2 | 1 | 3 | 1 | 1 | 1 | 0 | 0 | 0 | 0 | 1 | 2 |
| 8 | MG-132 | Ra 09-07 | 1 | 0 | 1 | 2 | 1 | 1 | 0 | 0 | 1 | 0 | 0 | 0 | 0 | 1 | 1 |
| 8 | MG-132 | Ra 10-42 | 1 | 0 | 0 | 0 | 0 | 0 | 0 | 0 | 0 | 0 | 1 | 0 | 0 | 1 | 1 |
| 8 | MG-132 | Ra 10-42 | 1 | 1 | 1 | 4 | 1 | 1 | 0 | 1 | 0 | 0 | 1 | 1 | 1 | 1 | 2 |
| 8 | MG-132 | Ra 10-42 | 1 | 1 | 1 | 2 | 1 | 1 | 0 | 1 | 0 | 0 | 1 | 0 | 0 | 1 | 2 |
| 8 | MG-132 | Ra 10-42 | 1 | 1 | 1 | 4 | 1 | 1 | 0 | 0 | 1 | 1 | 1 | 1 | 1 | 1 | 2 |
| 8 | MG-132 | Ra 10-42 | 1 | 1 | 1 | 2 | 0 | 0 | 0 | 0 | 0 | 0 | 0 | 0 | 0 | 1 | 2 |
| 8 | MG-132 | Ra 10-46 | 1 | 1 | 1 | 3 | 0 | 0 | 0 | 0 | 0 | 0 | 1 | 0 | 0 | 0 | 0 |
| 8 | MG-132 | Ra 10-46 | 1 | 1 | 1 | 3 | 1 | 1 | 0 | 1 | 0 | 0 | 1 | 0 | 0 | 1 | 1 |
| 8 | MG-132 | Ra 10-46 | 1 | 1 | 1 | 4 | 1 | 1 | 0 | 1 | 0 | 0 | 1 | 0 | 0 | 1 | 1 |
| 8 | MG-132 | Ra 10-46 | 1 | 1 | 1 | 3 | 1 | 2 | 0 | 2 | 0 | 0 | 0 | 1 | 1 | 1 | 2 |
| 8 | MG-132 | Ra 10-46 | 1 | 0 | 1 | 1 | 0 | 0 | 0 | 0 | 0 | 0 | 0 | 0 | 0 | 0 | 0 |
| 8 | Lac | Ra 10-47 | 1 | 0 | 1 | 2 | 1 | 2 | 0 | 1 | 0 | 1 | 1 | 0 | 0 | 1 | 2 |
| 8 | Lac | Ra 10-47 | 1 | 1 | 1 | 6 | 1 | 2 | 0 | 1 | 1 | 1 | 1 | 0 | 0 | 1 | 2 |
| 8 | Lac | Ra 10-47 | 1 | 1 | 1 | 4 | 1 | 3 | 1 | 1 | 0 | 1 | 1 | 0 | 0 | 1 | 2 |
| 8 | Lac | Ra 10-47 | 1 | 1 | 1 | 3 | 1 | 3 | 0 | 1 | 1 | 1 | 1 | 0 | 0 | 1 | 2 |
| 8 | Lac | Ra 10-47 | 1 | 1 | 1 | 1 | 1 | 2 | 0 | 1 | 1 | 0 | 0 | 0 | 0 | 1 | 2 |
| 8 | Lac | Ra 10-50 | 1 | 0 | 1 | 4 | 1 | 1 | 0 | 1 | 0 | 0 | 1 | 0 | 0 | 1 | 1 |
| 8 | Lac | Ra 10-50 | 1 | 1 | 1 | 3 | 0 | 0 | 0 | 0 | 0 | 0 | 1 | 0 | 0 | 1 | 2 |
| 8 | Lac | Ra 10-50 | 1 | 1 | 1 | 2 | 0 | 0 | 0 | 0 | 0 | 0 | 1 | 0 | 0 | 1 | 1 |
| 8 | Lac | Ra 10-50 | 1 | 1 | 1 | 5 | 1 | 2 | 0 | 1 | 0 | 0 | 0 | 0 | 0 | 1 | 2 |
| 8 | Lac | Ra 10-50 | 1 | 3 | 0 | 0 | 1 | 2 | 0 | 1 | 1 | 0 | 0 | 0 | 0 | 1 | 2 |
| 8 | Lac | Ra 10-51 | 1 | 0 | 1 | 2 | 0 | 0 | 0 | 0 | 0 | 0 | 0 | 0 | 0 | 0 | 0 |
| 8 | Lac | Ra 10-51 | 1 | 2 | 1 | 4 | 1 | 2 | 1 | 1 | 0 | 0 | 1 | 0 | 0 | 1 | 2 |
| 8 | Lac | Ra 10-51 | 1 | 1 | 1 | 7 | 1 | 2 | 1 | 1 | 0 | 0 | 1 | 0 | 0 | 1 | 2 |
| 8 | Lac | Ra 10-51 | 1 | 1 | 1 | 4 | 1 | 2 | 1 | 1 | 0 | 0 | 1 | 0 | 0 | 1 | 1 |
| 8 | Lac | Ra 10-51 | 1 | 0 | 1 | 3 | 1 | 2 | 0 | 1 | 1 | 0 | 0 | 0 | 0 | 1 | 2 |
| 8 | MG-132 | Ra 09-08 | 1 | 0 | 1 | 2 | 0 | 0 | 0 | 0 | 0 | 0 | 0 | 0 | 0 | 0 | 0 |
| 8 | MG-132 | Ra 09-08 | 1 | 1 | 1 | 4 | 1 | 1 | 0 | 1 | 0 | 1 | 1 | 0 | 0 | 1 | 1 |
| 8 | MG-132 | Ra 09-08 | 1 | 1 | 1 | 8 | 1 | 1 | 1 | 0 | 0 | 0 | 1 | 0 | 0 | 1 | 2 |
| 8 | MG-132 | Ra 09-08 | 1 | 1 | 1 | 5 | 1 | 2 | 1 | 1 | 0 | 0 | 1 | 0 | 0 | 1 | 2 |
| 8 | MG-132 | Ra 09-08 | 1 | 1 | 1 | 8 | 1 | 2 | 0 | 1 | 1 | 0 | 0 | 0 | 0 | 1 | 2 |
| 8 | Lac | Ra 10-48 | 1 | 0 | 1 | 2 | 1 | 2 | 0 | 1 | 0 | 1 | 1 | 0 | 0 | 1 | 1 |
| 8 | Lac | Ra 10-48 | 1 | 1 | 1 | 6 | 1 | 2 | 0 | 1 | 1 | 1 | 1 | 0 | 0 | 1 | 2 |
| 8 | Lac | Ra 10-48 | 1 | 1 | 1 | 3 | 1 | 3 | 1 | 1 | 0 | 1 | 1 | 0 | 0 | 1 | 2 |
| 8 | Lac | Ra 10-48 | 1 | 1 | 1 | 4 | 1 | 3 | 0 | 1 | 1 | 1 | 1 | 0 | 0 | 1 | 2 |
| 8 | Lac | Ra 10-48 | 1 | 1 | 1 | 2 | 1 | 2 | 0 | 1 | 1 | 0 | 0 | 0 | 0 | 1 | 2 |
| 8 | Lac | Ra 10-49 | 1 | 0 | 1 | 4 | 1 | 1 | 0 | 1 | 0 | 0 | 1 | 0 | 0 | 1 | 1 |
| 8 | Lac | Ra 10-49 | 1 | 1 | 1 | 5 | 0 | 0 | 0 | 0 | 0 | 0 | 1 | 0 | 0 | 1 | 1 |
| 8 | Lac | Ra 10-49 | 1 | 1 | 1 | 2 | 0 | 0 | 0 | 0 | 0 | 0 | 1 | 0 | 0 | 1 | 2 |
| 8 | Lac | Ra 10-49 | 1 | 1 | 1 | 4 | 1 | 2 | 0 | 1 | 0 | 0 | 0 | 0 | 0 | 1 | 2 |
| 8 | Lac | Ra 10-49 | 1 | 3 | 0 | 0 | 1 | 2 | 0 | 1 | 1 | 0 | 0 | 0 | 0 | 1 | 2 |
| 2 | DMSO | Ra 11-73 | 1 | 0 | 0 | 0 | 0 | 0 | 0 | 0 | 0 | 0 | 0 | 0 | 0 | 0 | 0 |
| 2 | DMSO | Ra 11-73 | 1 | 0 | 0 | 0 | 0 | 0 | 0 | 0 | 0 | 0 | 0 | 0 | 0 | 0 | 0 |
| 2 | DMSO | Ra 11-73 | 1 | 1 | 0 | 0 | 0 | 0 | 0 | 0 | 0 | 0 | 0 | 0 | 0 | 0 | 0 |
| 2 | DMSO | Ra 11-73 | 1 | 1 | 1 | 4 | 1 | 2 | 1 | 1 | 0 | 0 | 0 | 1 | 2 | 1 | 2 |
| 2 | DMSO | Ra 11-73 | 1 | 0 | 0 | 0 | 1 | 1 | 0 | 1 | 0 | 0 | 0 | 0 | 0 | 1 | 3 |
| 2 | DMSO | Ra 11-74 | 1 | 0 | 0 | 0 | 0 | 0 | 0 | 0 | 0 | 0 | 0 | 0 | 0 | 0 | 0 |
| 2 | DMSO | Ra 11-74 | 1 | 0 | 1 | 1 | 0 | 0 | 0 | 0 | 0 | 0 | 0 | 0 | 0 | 0 | 0 |
| 2 | DMSO | Ra 11-74 | 1 | 1 | 1 | 3 | 0 | 0 | 0 | 0 | 0 | 0 | 1 | 0 | 0 | 1 | 1 |
| 2 | DMSO | Ra 11-74 | 1 | 0 | 1 | 3 | 0 | 0 | 0 | 0 | 0 | 0 | 0 | 0 | 0 | 1 | 1 |
| 2 | DMSO | Ra 11-74 | 1 | 1 | 1 | 1 | 1 | 1 | 0 | 1 | 0 | 0 | 0 | 0 | 0 | 0 | 0 |
| 2 | MG-132 | Ra 11-78 | 1 | 0 | 1 | 5 | 1 | 2 | 1 | 1 | 0 | 1 | 1 | 0 | 0 | 1 | 1 |
| 2 | MG-132 | Ra 11-78 | 1 | 0 | 1 | 7 | 1 | 1 | 0 | 1 | 0 | 1 | 1 | 1 | 1 | 1 | 2 |
| 2 | MG-132 | Ra 11-78 | 1 | 0 | 1 | 3 | 1 | 1 | 0 | 1 | 0 | 1 | 1 | 0 | 0 | 1 | 2 |
| 2 | MG-132 | Ra 11-78 | 1 | 1 | 1 | 3 | 1 | 3 | 1 | 1 | 0 | 0 | 0 | 0 | 0 | 1 | 3 |
| 2 | MG-132 | Ra 11-78 | 1 | 1 | 1 | 4 | 1 | 2 | 1 | 1 | 0 | 0 | 0 | 0 | 0 | 1 | 2 |
| 2 | MG-132 | Ra 11-79 | 1 | 0 | 1 | 1 | 0 | 0 | 0 | 0 | 0 | 0 | 0 | 0 | 0 | 0 | 0 |
| 2 | MG-132 | Ra 11-79 | 1 | 0 | 1 | 4 | 1 | 1 | 0 | 1 | 0 | 0 | 0 | 0 | 0 | 0 | 0 |
| 2 | MG-132 | Ra 11-79 | 1 | 1 | 1 | 3 | 1 | 3 | 1 | 1 | 0 | 0 | 1 | 0 | 0 | 1 | 2 |
| 2 | MG-132 | Ra 11-79 | 1 | 1 | 1 | 5 | 1 | 2 | 1 | 1 | 0 | 0 | 1 | 0 | 0 | 1 | 3 |
| 2 | MG-132 | Ra 11-79 | 1 | 1 | 1 | 2 | 1 | 2 | 0 | 1 | 1 | 0 | 0 | 0 | 0 | 1 | 3 |
| 2 | Lac | Ra 11-80 | 1 | 0 | 0 | 0 | 0 | 0 | 0 | 0 | 0 | 0 | 0 | 1 | 1 | 0 | 0 |
| 2 | Lac | Ra 11-80 | 1 | 0 | 1 | 3 | 1 | 1 | 0 | 1 | 0 | 0 | 1 | 0 | 0 | 1 | 1 |
| 2 | Lac | Ra 11-80 | 1 | 1 | 1 | 5 | 1 | 2 | 1 | 1 | 0 | 0 | 1 | 1 | 1 | 1 | 2 |
| 2 | Lac | Ra 11-80 | 1 | 1 | 1 | 3 | 1 | 1 | 0 | 1 | 0 | 0 | 1 | 0 | 0 | 1 | 3 |
| 2 | Lac | Ra 11-80 | 1 | 0 | 1 | 2 | 1 | 1 | 1 | 0 | 0 | 0 | 0 | 0 | 0 | 1 | 2 |
| 2 | Lac | Ra 11-82 | 1 | 0 | 0 | 0 | 0 | 0 | 0 | 0 | 0 | 0 | 0 | 0 | 0 | 0 | 0 |
| 2 | Lac | Ra 11-82 | 1 | 1 | 1 | 4 | 1 | 2 | 1 | 1 | 0 | 0 | 0 | 0 | 0 | 1 | 1 |
| 2 | Lac | Ra 11-82 | 1 | 0 | 1 | 5 | 1 | 1 | 1 | 1 | 0 | 0 | 1 | 0 | 0 | 1 | 2 |
| 2 | Lac | Ra 11-82 | 1 | 1 | 1 | 4 | 1 | 2 | 0 | 1 | 0 | 0 | 1 | 0 | 0 | 1 | 3 |
| 2 | Lac | Ra 11-82 | 1 | 1 | 1 | 4 | 1 | 3 | 1 | 1 | 0 | 0 | 0 | 0 | 0 | 1 | 3 |
| 2 | Epox | Ra 11-88 | 1 | 0 | 1 | 3 | 0 | 0 | 0 | 0 | 0 | 0 | 0 | 0 | 0 | 0 | 0 |
| 2 | Epox | Ra 11-88 | 1 | 1 | 1 | 7 | 1 | 3 | 0 | 1 | 0 | 0 | 1 | 0 | 0 | 1 | 2 |
| 2 | Epox | Ra 11-88 | 1 | 1 | 1 | 5 | 1 | 3 | 1 | 1 | 0 | 0 | 1 | 1 | 1 | 1 | 3 |
| 2 | Epox | Ra 11-88 | 1 | 1 | 1 | 9 | 1 | 3 | 1 | 1 | 0 | 0 | 1 | 1 | 1 | 1 | 3 |
| 2 | Epox | Ra 11-88 | 1 | 1 | 1 | 6 | 1 | 3 | 0 | 1 | 1 | 0 | 1 | 0 | 0 | 1 | 3 |
| 2 | Epox | Ra 11-89 | 1 | 0 | 0 | 0 | 1 | 2 | 0 | 1 | 0 | 0 | 1 | 1 | 1 | 1 | 2 |
| 2 | Epox | Ra 11-89 | 1 | 1 | 0 | 0 | 1 | 3 | 1 | 1 | 0 | 0 | 1 | 0 | 0 | 1 | 3 |
| 2 | Epox | Ra 11-89 | 1 | 1 | 1 | 7 | 1 | 3 | 0 | 1 | 0 | 0 | 1 | 0 | 0 | 1 | 3 |
| 2 | Epox | Ra 11-89 | 1 | 1 | 1 | 6 | 1 | 3 | 0 | 1 | 0 | 0 | 0 | 1 | 1 | 1 | 2 |
| 2 | Epox | Ra 11-89 | 1 | 1 | 1 | 5 | 1 | 2 | 0 | 1 | 1 | 0 | 0 | 0 | 0 | 1 | 2 |

## Statistical analysis

The statistical analysis was performed using the data analysis software system STATISTICA version 12, StatSoft Inc. (2014).

The frequency of ependymal atrophy, apoptosis, ependymal discontinuity and rosettes, glial nodules and inflammatory cell infiltration in the animals from the various experimental groups sacrificed 2 weeks and 8 weeks after studied substances application was calculated. A 2x2 Fisher test was utilized to compare these frequencies for each substance. The level of significance was set to 0.05. The differences between the control (DMSO) group and each of the other experimental groups (MG-132 or lactacystin or epoxomicin) were also compared at 2 weeks and 8 weeks using the 2x2 Fisher test by taking into account the experiment-wise error rate and applying Šidak’s correction [[2](#_ENREF_2)] for multiple comparisons. The level of significance was set to 0.017.

As an example of this calculation statistic analysis of the frequency of ependymal atrophy is shown:

**Statistics Atrophy**

**:**

|  | atrophy |  |  |  |  |
| --- | --- | --- | --- | --- | --- |
| DMSO | present | absent |  |  |  |
| 2 week | 7 | 18 |  |  |  |
| 8 week | 13 | 12 |  |  |  |
| Test of Independence in Contingency Table | | | |  |  |
| Frequency Table (Upper: Observed; Lower: Expected) | | | | |  |
|  |  |  |  |  |  |
|  | present | absent | Total |  |  |
| 2 week | 7 | 18 | 25 |  |  |
|  | 10 | 15 |  |  |  |
| 8 week | 13 | 12 | 25 |  |  |
|  | 10 | 15 |  |  |  |
| Total | 20 | 30 | 50 |  |  |
|  |  |  |  |  |  |
| Chi-square Test | |  |  |  |  |
| Degrees of freedom | | 1 |  |  |  |
| Chi^2 (no Correction) | | 3 | N.S. (P>0.05) (two-sided) | | |
| P (two-sided) | | 0,083265 |  |  |  |
| Chi^2 (Yates' correction) | | 2,083333 | N.S. (P>0.05) (two-sided) | | |
| P (two-sided) | | 0,148915 |  |  |  |
|  |  |  |  |  |  |
| Phi |  | 0,244949 |  |  |  |
| Phi^2 |  | 0,06 |  |  |  |
|  |  |  |  |  |  |
| Likelhood Ratio Test | |  |  |  |  |
| Degrees of freedom | | 1 |  |  |  |
| G^2 (Likelhood Ratio) | | 3,036152 | N.S. (P>0.05) (two-sided) | | |
| P (two-sided) | | 0,081429 |  |  |  |
|  |  |  |  |  |  |
| Fisher's Exact Probability | | |  |  |  |
| P (one-sided) | | 0,07408 | N.S. (P>0.05) one-sided | | |
|  |  |  |  |  |  |

|  | atrophy |  |  |  |  |
| --- | --- | --- | --- | --- | --- |
| Epox | present | absent |  |  |  |
| 2 week | 21 | 4 |  |  |  |
| 8 week | 16 | 9 |  |  |  |
|  |  |  |  |  |  |
|  |  |  |  |  |  |
| Test of Independence in Contingency Table | | | |  |  |
|  |  |  |  |  |  |
| Frequency Table (Upper: Observed; Lower: Expected) | | | | |  |
|  |  |  |  |  |  |
|  | present | absent | Total |  |  |
| 2 week | 21 | 4 | 25 |  |  |
|  | 18,5 | 6,5 |  |  |  |
| 8 week | 16 | 9 | 25 |  |  |
|  | 18,5 | 6,5 |  |  |  |
| Total | 37 | 13 | 50 |  |  |
|  |  |  |  |  |  |
| Chi-square Test | |  |  |  |  |
| Degrees of freedom | | 1 |  |  |  |
| Chi^2 (no Correction) | | 2,598753 | N.S. (P>0.05) (two-sided) | | |
| P (two-sided) | | 0,106948 |  |  |  |
| Chi^2 (Yates' correction) | | 1,663202 | N.S. (P>0.05) (two-sided) | | |
| P (two-sided) | | 0,197172 |  |  |  |
|  |  |  |  |  |  |
| Phi |  | 0,22798 |  |  |  |
| Phi^2 |  | 0,051975 |  |  |  |
|  |  |  |  |  |  |
| Likelhood Ratio Test | |  |  |  |  |
| Degrees of freedom | | 1 |  |  |  |
| G^2 (Likelhood Ratio) | | 2,651288 | N.S. (P>0.05) (two-sided) | | |
| P (two-sided) | | 0,103466 |  |  |  |
|  |  |  |  |  |  |
| Fisher's Exact Probability | | |  |  |  |
| P (one-sided) | | 0,098164 | N.S. (P>0.05) one-sided | | |
|  |  |  |  |  |  |
|  | atrophy |  |  |  |  |
| mg132 | present | absent |  |  |  |
| 2 week | 12 | 13 |  |  |  |
| 8 week | 24 | 1 |  |  |  |
|  |  |  |  |  |  |
|  |  |  |  |  |  |
| Test of Independence in Contingency Table |  |  |  |  |  |
|  |  |  |  |  |  |
| Frequency Table (Upper: Observed; Lower: Expected) |  |  |  |  |  |
|  |  |  |  |  |  |
|  | present | absent | Total |  |  |
| 2 week | 12 | 13 | 25 |  |  |
|  | 18 | 7 |  |  |  |
| 8 week | 24 | 1 | 25 |  |  |
|  | 18 | 7 |  |  |  |
| Total | 36 | 14 | 50 |  |  |
|  |  |  |  |  |  |
| Chi-square Test |  |  |  |  |  |
| Degrees of freedom |  | 1 |  |  |  |
| Chi^2 (no Correction) |  | 14,28571 | *** (P<=0.001) (two-sided) |  |  |
| P (two-sided) |  | 0,000157 |  |  |  |
| Chi^2 (Yates' correction) |  | 12,00397 | *** (P<=0.001) (two-sided) |  |  |
| P (two-sided) |  | 0,000531 |  |  |  |
|  |  |  |  |  |  |
| Phi |  | 0,534522 |  |  |  |
| Phi^2 |  | 0,285714 |  |  |  |
|  |  |  |  |  |  |
| Likelhood Ratio Test |  |  |  |  |  |
| Degrees of freedom |  | 1 |  |  |  |
| G^2 (Likelhood Ratio) |  | 16,28078 | *** (P<=0.001) (two-sided) |  |  |
| P (two-sided) |  | 5,46E-05 |  |  |  |
|  |  |  |  |  |  |
| Fisher's Exact Probability |  |  |  |  |  |
| P (one-sided) |  | 0,000144 | *** (P<=0.001) one-sided |  |  |
|  |  |  |  |  |  |
| 2 weeks |  | p<0,016952 | |  |  |
|  |  |  |  |  |  |
|  | present | absent |  |  |  |
| DMSO | 7 | 18 |  |  |  |
| MG | 13 | 12 |  |  |  |
|  |  |  |  |  |  |
|  |  |  |  |  |  |
| Test of Independence in Contingency Table | | | |  |  |
|  |  |  |  |  |  |
| Frequency Table (Upper: Observed; Lower: Expected) | | | | |  |
|  |  |  |  |  |  |
|  | jest | nie ma | Total |  |  |
| D\|MSO | 7 | 18 | 25 |  |  |
|  | 10 | 15 |  |  |  |
| MG | 13 | 12 | 25 |  |  |
|  | 10 | 15 |  |  |  |
| Total | 20 | 30 | 50 |  |  |
|  |  |  |  |  |  |
| Chi-square Test | |  |  |  |  |
| Degrees of freedom | | 1 |  |  |  |
| Chi^2 (no Correction) | | 3 | N.S. (P>0.05) (two-sided) | |  |
| P (two-sided) | | 0,083265 |  |  |  |
| Chi^2 (Yates' correction) | | 2,083333 | N.S. (P>0.05) (two-sided) | |  |
| P (two-sided) | | 0,148915 |  |  |  |
|  |  |  |  |  |  |
| Phi |  | 0,244949 |  |  |  |
| Phi^2 |  | 0,06 |  |  |  |
|  |  |  |  |  |  |
| Likelhood Ratio Test | |  |  |  |  |
| Degrees of freedom | | 1 |  |  |  |
| G^2 (Likelhood Ratio) | | 3,036152 | N.S. (P>0.05) (two-sided) | |  |
| P (two-sided) | | 0,081429 |  |  |  |
|  |  |  |  |  |  |
| Fisher's Exact Probability | | |  |  |  |
| P (one-sided) | | 0,07408 | N.S. (P>0.05) one-sided | |  |
| 2 weeks |  | p<0,016952 | |  |  |
|  |  |  |  |  |  |
|  | present | absent |  |  |  |
| DMSO | 7 | 18 |  |  |  |
| Epoxy | 21 | 4 |  |  |  |
|  |  |  |  |  |  |
|  |  |  |  |  |  |
| Test of Independence in Contingency Table | | | |  |  |
|  |  |  |  |  |  |
| Frequency Table (Upper: Observed; Lower: Expected) | | | | |  |
|  |  |  |  |  |  |
|  | jest | nie ma | Total |  |  |
| DMSO | 7 | 18 | 25 |  |  |
|  | 14 | 11 |  |  |  |
| Epoxy | 21 | 4 | 25 |  |  |
|  | 14 | 11 |  |  |  |
| Total | 28 | 22 | 50 |  |  |
|  |  |  |  |  |  |
| Chi-square Test | |  |  |  |  |
| Degrees of freedom | | 1 |  |  |  |
| Chi^2 (no Correction) | | 15,90909 | *** (P<=0.001) (two-sided) | |  |
| P (two-sided) | | 6,65E-05 |  |  |  |
| Chi^2 (Yates' correction) | | 13,71753 | *** (P<=0.001) (two-sided) | |  |
| P (two-sided) | | 0,000212 |  |  |  |
|  |  |  |  |  |  |
| Phi |  | 0,564076 |  |  |  |
| Phi^2 |  | 0,318182 |  |  |  |
|  |  |  |  |  |  |
| Likelhood Ratio Test | |  |  |  |  |
| Degrees of freedom | | 1 |  |  |  |
| G^2 (Likelhood Ratio) | | 16,96182 | *** (P<=0.001) (two-sided) | |  |
| P (two-sided) | | 3,81E-05 |  |  |  |
|  |  |  |  |  |  |
| Fisher's Exact Probability | | |  |  |  |
| P (one-sided) | | 7,33E-05 | *** (P<=0.001) one-Sided | |  |

| 8 weeks |  | p<0,016952 | |  |  |
| --- | --- | --- | --- | --- | --- |
|  |  |  |  |  |  |
|  | present | absent |  |  |  |
| DMSO | 13 | 12 |  |  |  |
| Lac | 24 | 1 |  |  |  |
|  |  |  |  |  |  |
|  |  |  |  |  |  |
| Test of Independence in Contingency Table | | | |  |  |
|  |  |  |  |  |  |
| Frequency Table (Upper: Observed; Lower: Expected) | | | | |  |
|  |  |  |  |  |  |
|  | jest | nie ma | Total |  |  |
| DMSO | 13 | 12 | 25 |  |  |
|  | 18,5 | 6,5 |  |  |  |
| Lac | 24 | 1 | 25 |  |  |
|  | 18,5 | 6,5 |  |  |  |
| Total | 37 | 13 | 50 |  |  |
|  |  |  |  |  |  |
| Chi-square Test | |  |  |  |  |
| Degrees of freedom | | 1 |  |  |  |
| Chi^2 (no Correction) | | 12,57796 | *** (P<=0.001) (two-sided) | | |
| P (two-sided) | | 0,00039 |  |  |  |
| Chi^2 (Yates' correction) | | 10,39501 | ** (P<=0.01) (two-sided) | | |
| P (two-sided) | | 0,001264 |  |  |  |
|  |  |  |  |  |  |
| Phi |  | 0,501557 |  |  |  |
| Phi^2 |  | 0,251559 |  |  |  |
|  |  |  |  |  |  |
| Likelhood Ratio Test | |  |  |  |  |
| Degrees of freedom | | 1 |  |  |  |
| G^2 (Likelhood Ratio) | | 14,29114 | *** (P<=0.001) (two-sided) | | |
| P (two-sided) | | 0,000157 |  |  |  |
|  |  |  |  |  |  |
| Fisher's Exact Probability | | |  |  |  |
| P (one-sided) | | 0,000381 | *** (P<=0.001) one-sided | | |

The distributions of the ependymal discontinuity and mononuclear inflammatory cell infiltration scores were compared. The differences between the control (DMSO) group and each of the other experimental groups (MG-132 or lactacystin or epoxomicin) were compared at 2 weeks and 8 weeks using the Chi-square test, also taking into account the experiment-wise error rate and applying Šidak’s correction for multiple comparisons. The level of significance was set to 0.017. As an example we present below calculation for the distributions of the mononuclear inflammatory cell infiltration scores after two weeks.

|  |  |  |  |  |  |  |  |
| --- | --- | --- | --- | --- | --- | --- | --- |
| **Data Entry Section:** | |  |  |  |  |  |  |
| **Enter Desired α Level:** | | 0,016952 |  |  |  | | |
|  |  |  |  |  |  |  |  |
| **Observed Matrix:** | |  |  |  |  |  |  |
| **Row** | **DMSO** | **MG132** | **Col 3** | **Col 4** | **Col 5** | **Col 6** | **Totals** |
| **1** | 14 | 4 |  |  |  |  | **18** |
| **2** | 3 | 2 |  |  |  |  | **5** |
| **3** | 5 | 12 |  |  |  |  | **17** |
| **4** | 3 | 7 |  |  |  |  | **10** |
| **5** |  |  |  |  |  |  | **0** |
| **6** |  |  |  |  |  |  | **0** |
| **Totals** | **25** | **25** | **0** | **0** | **0** | **0** | **50** |
|  |  |  |  |  |  |  |  |
| **Output Section:** | |  |  | **χ^2^=** | **10,238** |  |  |
|  |  |  |  | **DF=** | **3** |  |  |
|  |  |  |  | **Crit. χ^2^=** | **10,199** |  |  |
|  |  |  |  | **Decision** | **Reject Ho** |  |  |
|  |  |  |  | **Prob=** | **0,017** |  |  |
| **Expected Matrix [Summary]:** | | |  |  |  |  |  |
| **Row** | **DMSO** | **MG132** | **Col 3** | **Col 4** | **Col 5** | **Col 6** | **Totals** |
| **1** | 9,000 | 9,000 |  |  |  |  | **18** |
| **2** | 2,500 | 2,500 |  |  |  |  | **5** |
| **3** | 8,500 | 8,500 |  |  |  |  | **17** |
| **4** | 5,000 | 5,000 |  |  |  |  | **10** |
| **5** |  |  |  |  |  |  | **0** |
| **6** |  |  |  |  |  |  | **0** |
| **Totals** | **25** | **25** | **0** | **0** | **0** | **0** | **50** |
|  |  |  |  |  |  |  |  |
| **Chisquared Contribution Matrix [Summary]:** | | | | |  |  |  |
| **Row** | **DMSO** | **MG-132** | **Col 3** | **Col 4** | **Col 5** | **Col 6** | **Totals** |
| **1** | 2,778 | 2,778 |  |  |  |  | **5,556** |
| **2** | 0,100 | 0,100 |  |  |  |  | **0,200** |
| **3** | 1,441 | 1,441 |  |  |  |  | **2,882** |
| **4** | 0,800 | 0,800 |  |  |  |  | **1,600** |
| **5** |  |  |  |  |  |  | **0,000** |
| **6** |  |  |  |  |  |  | **0,000** |
| **Totals** | **5,119** | **5,119** | **0,000** | **0,000** | **0,000** | **0,000** | **10,238** |
|  |  |  |  |  |  |  |  |

| **Data Entry Section:** | | |  |  |  |  |  |
| --- | --- | --- | --- | --- | --- | --- | --- |
| **Enter Desired α Level:** | | 0,016952 |  |  |  | | |
|  |  |  |  |  |  |  |  |
| **Observed Matrix:** | |  |  |  |  |  |  |
| **Row** | **DMSO** | **Lactacystin** | **Col 3** | **Col 4** | **Col 5** | **Col 6** | **Totals** |
| **1** | 14 | 6 |  |  |  |  | **20** |
| **2** | 3 | 4 |  |  |  |  | **7** |
| **3** | 5 | 7 |  |  |  |  | **12** |
| **4** | 3 | 8 |  |  |  |  | **11** |
| **5** |  |  |  |  |  |  | **0** |
| **6** |  |  |  |  |  |  | **0** |
| **Totals** | **25** | **25** | **0** | **0** | **0** | **0** | **50** |
|  |  |  |  |  |  |  |  |
| **Output Section:** | |  |  | **χ^2^=** | **5,949** |  |  |
|  |  |  |  | **DF=** | **3** |  |  |
|  |  |  |  | **Crit. χ^2^=** | **10,199** |  |  |
|  |  |  |  | **Decision** | **Accept Ho** |  |  |
|  |  |  |  | **Prob=** | **0,114** |  |  |
| **Expected Matrix [Summary]:** | | |  |  |  |  |  |
| **Row** | **DMSO** | **Lactacystin** | **Col 3** | **Col 4** | **Col 5** | **Col 6** | **Totals** |
| **1** | 10,000 | 10,000 |  |  |  |  | **20** |
| **2** | 3,500 | 3,500 |  |  |  |  | **7** |
| **3** | 6,000 | 6,000 |  |  |  |  | **12** |
| **4** | 5,500 | 5,500 |  |  |  |  | **11** |
| **5** |  |  |  |  |  |  | **0** |
| **6** |  |  |  |  |  |  | **0** |
| **Totals** | **25** | **25** | **0** | **0** | **0** | **0** | **50** |
|  |  |  |  |  |  |  |  |
| **Chisquared Contribution Matrix [Summary]:** | | | | |  |  |  |
| **Row** | **DMSO** | **Lactacystin** | **Col 3** | **Col 4** | **Col 5** | **Col 6** | **Totals** |
| **1** | 1,600 | 1,600 |  |  |  |  | **3,200** |
| **2** | 0,071 | 0,071 |  |  |  |  | **0,143** |
| **3** | 0,167 | 0,167 |  |  |  |  | **0,333** |
| **4** | 1,136 | 1,136 |  |  |  |  | **2,273** |
| **5** |  |  |  |  |  |  | **0,000** |
| **6** |  |  |  |  |  |  | **0,000** |
| **Totals** | **2,974** | **2,974** | **0,000** | **0,000** | **0,000** | **0,000** | **5,949** |
|  |  |  |  |  |  |  |  |

| **Data Entry Section:** | |  |  |  |  |  |  |
| --- | --- | --- | --- | --- | --- | --- | --- |
| **Enter Desired α Level:** | | 0,016952 |  |  |  | | |
|  |  |  |  |  |  |  |  |
| **Observed Matrix:** | |  |  |  |  |  |  |
| **Row** | **DMSO** | **Epoxomicin** | **Col 3** | **Col 4** | **Col 5** | **Col 6** | **Totals** |
| **1** | 14 | 2 |  |  |  |  | **16** |
| **2** | 3 | 0 |  |  |  |  | **3** |
| **3** | 5 | 9 |  |  |  |  | **14** |
| **4** | 3 | 14 |  |  |  |  | **17** |
| **5** |  |  |  |  |  |  | **0** |
| **6** |  |  |  |  |  |  | **0** |
| **Totals** | **25** | **25** | **0** | **0** | **0** | **0** | **50** |
|  |  |  |  |  |  |  |  |
| **Output Section:** | |  |  | **χ^2^=** | **20,261** |  |  |
|  |  |  |  | **DF=** | **3** |  |  |
|  |  |  |  | **Crit. χ^2^=** | **10,199** |  |  |
|  |  |  |  | **Decision** | **Reject Ho** |  |  |
|  |  |  |  | **Prob=** | **0,000** |  |  |
| **Expected Matrix [Summary]:** | | |  |  |  |  |  |
| **Row** | **DMSO** | **Epoxomicin** | **Col 3** | **Col 4** | **Col 5** | **Col 6** | **Totals** |
| **1** | 8,000 | 8,000 |  |  |  |  | **16** |
| **2** | 1,500 | 1,500 |  |  |  |  | **3** |
| **3** | 7,000 | 7,000 |  |  |  |  | **14** |
| **4** | 8,500 | 8,500 |  |  |  |  | **17** |
| **5** |  |  |  |  |  |  | **0** |
| **6** |  |  |  |  |  |  | **0** |
| **Totals** | **25** | **25** | **0** | **0** | **0** | **0** | **50** |
|  |  |  |  |  |  |  |  |
| **Chisquared Contribution Matrix [Summary]:** | | | | |  |  |  |
| **Row** | **DMSO** | **Epoxomicin** | **Col 3** | **Col 4** | **Col 5** | **Col 6** | **Totals** |
| **1** | 4,500 | 4,500 |  |  |  |  | **9,000** |
| **2** | 1,500 | 1,500 |  |  |  |  | **3,000** |
| **3** | 0,571 | 0,571 |  |  |  |  | **1,143** |
| **4** | 3,559 | 3,559 |  |  |  |  | **7,118** |
| **5** |  |  |  |  |  |  | **0,000** |
| **6** |  |  |  |  |  |  | **0,000** |
| **Totals** | **10,130** | **10,130** | **0,000** | **0,000** | **0,000** | **0,000** | **20,261** |

The differences in (1) the number of apoptotic bodies per section and (2) the length of the adhesions connecting the ventricular walls were compared between the experimental groups using two-way ANOVA, followed by Fisher’s post-hoc LSD test (upon normality checking using the Kolmogorov-Smirnov test). The differences were considered significant when p<0.05. As an example statistic analysis of the number of apoptotic bodies per section is present.

References

1. Paxinos G, Watson C. The rat brain in stereotaxic coordinates. 4th ed. ed. San Diego ; London: Academic; 1998.

2. Abdi H. The Bonferonni and Šidák Corrections for Multiple Comparisons. In: Salkind NJ, editor. Encyclopedia of Measurement and Statistics. Thousand Oaks, CA: SAGE Publications, Inc.; 2007. p. 103-7.
